# Characterization of *Staphylococcus aureus* CC1 and CC1660 of Human and Equine Origin

**DOI:** 10.3390/antibiotics14111082

**Published:** 2025-10-27

**Authors:** Johanna Jahnen, Christiane Cuny, Wolfgang Witte, Ralf Ehricht, Stefan Monecke, Dennis Hanke, Tanja Ahrens, Marta Leal, Sofia S. Costa, Isabel Couto, Stefan Schwarz, Andrea T. Feßler

**Affiliations:** 1 Institute of Microbiology and Epizootics, Centre for Infection Medicine, School of Veterinary Medicine, Freie Universität Berlin, 14163 Berlin, Germany; johanna.jahnen@fu-berlin.de (J.J.); dennis.hanke@fu-berlin.de (D.H.); tanja.ahrens@fu-berlin.de (T.A.); 2 Veterinary Centre for Resistance Research (TZR), School of Veterinary Medicine, Freie Universität Berlin, 14163 Berlin, Germany; 3 Robert Koch Institute, 38855 Wernigerode, Germany; cunych@rki.de (C.C.); ewwitte@t-online.de (W.W.); 4 Leibniz Institute of Photonic Technology, Member of the Research Alliance “Leibniz Health Technologies” and the Leibniz Centre for Photonics in Infection Research (LPI), 07745 Jena, Germany; ralf.ehricht@leibniz-ipht.de (R.E.); stefan.monecke@leibniz-ipht.de (S.M.); 5 InfectoGnostics Research Campus Jena, Center for Applied Research, 07743 Jena, Germany; 6 Institute of Physical Chemistry, Friedrich Schiller University Jena, 07743 Jena, Germany; 7 Global Health and Tropical Medicine (GHTM), LA-REAL, Instituto de Higiene e Medicina Tropical (IHMT), Universidade NOVA de Lisboa, 1349-008 Lisbon, Portugal; a21001253@ihmt.unl.pt (M.L.); scosta@ihmt.unl.pt (S.S.C.); icouto@ihmt.unl.pt (I.C.)

**Keywords:** horse-associated *Staphylococcus aureus* lineage, whole-genome sequencing, cgMLST, MLST, *arcC* deletion, antimicrobial susceptibility testing, antimicrobial resistance patterns, *lukP/lukQ* (=*lukPQ*), horse-specific leukocidin

## Abstract

**Background/Objectives**: *Staphylococcus aureus* isolates from humans and horses of the equine-associated clonal complexes (CCs) CC1 and CC1660 were comparatively investigated for their genomic relationships. **Methods**: A total of 91 *S. aureus* isolates (64 human, 27 equine) were subjected to whole-genome sequencing (WGS), sequence analysis, and antimicrobial susceptibility testing. **Results**: WGS confirmed 75 CC1 and 16 CC1660 isolates, comprising nine sequence types (STs) in CC1 and four STs in CC1660. Ten *spa* types were present in CC1 and five in CC1660. In the *arcC* gene of three CC1 isolates, a 285 bp deletion was detected, and a nucleotide deletion causing a premature stop codon was found in one CC1660 isolate. Core genome (cg) MLST revealed a minimum difference of 1398/1492 alleles between the two CCs. All CC1 isolates harbored *agr* group III and capsule type 8 alleles, whereas all CC1660 isolates had *agr* group II and capsule type 5 alleles. Antimicrobial susceptibility testing revealed 18 phenotypic and 19 genotypic resistance patterns. All isolates were susceptible to vancomycin, linezolid and quinupristin–dalfopristin. Several virulence genes were detected in different combinations. The equine leukocidin genes *lukP/lukQ* were found in 22 isolates from horses and 38 isolates from humans, of which 35 had confirmed contact with horses. No Panton–Valentine leukocidin genes were found. Three human CC1660 isolates carried the toxic shock syndrome toxin-1 gene *tst-1*. **Conclusions**: The analysis of the 91 isolates might suggest intra- and interspecies transmission among and between humans and horses, which should be monitored in the future.

## 1. Introduction

*Staphylococcus aureus* (*S. aureus*) is mainly a harmless colonizer of humans and animals, but it can also cause severe infections in cases of predisposition. It is involved in several infections, including wound infections, skin and soft tissue infections, dermatitis, infective endocarditis, pneumonia, bacteremia, sepsis, osteomyelitis and septic arthritis in humans [1]. This pathogen is also capable of inducing similar infections in animals with notable examples being bovine mastitis [2] and wound infections affecting various animal species [3], including horses [4].

To facilitate the phylogenetic classification of *S. aureus*, Multilocus Sequence Typing (MLST) was established based on sequence variability within seven housekeeping genes [5]. MLST assigns isolates to sequence types (STs), which are further grouped into clonal complexes (CCs) [6,7,8]. Interestingly, these CCs usually share common characteristics, which may include partial host specificity. For example, CC5, CC15, CC30, CC45, and CC121 are mainly human-associated [9,10,11], while mainly animal-associated CCs include CC49 (rodents) [12], CC692 (birds) [13,14], CC425 (lagomorphs, badgers and ruminants) [14,15,16,17], CC133 and CC522 (goats and sheep) [18,19], and CC479 and CC705 (cattle) [20]. However, host range is not strictly defined by the MLST genes themselves, but rather by broader features of the genome, including mobile genetic elements [21]. In comparison, other CCs, such as CC398, display a broad host spectrum and can be found in humans [22], pigs [22], cattle [23], horses [24] and poultry [25], among others.

Several *S. aureus* clonal complexes have been described among horses, including for example CC1, CC8, CC22, CC130, CC398 and CC1660 [26]. They were associated with various infections, such as soft tissue and joint infections, pneumonia, sinusitis, metritis, omphalophlebitis or wound infections [26]. Horses can be infected by human *S. aureus* strains and some of these strains spread nosocomially in horse clinics. Examples are a CC8 methicillin-resistant *S. aureus* (MRSA) strain that was brought from Southern Africa into Australia [27] and the “Hannover Epidemic Strain”, another nosocomial CC8 MRSA that was common in Germany during the 1990s in humans and during the 2000s in horses [26]. Among horses, other ST8 isolates have also been found, including, e.g., USA500 and/or the Canadian epidemic MRSA lineage CMRSA-5 with *spa* type t064 and SCC*mec*IV elements, which was not present in our collection [28]. This might also be true for CC22 as CC22-MRSA-IV occurs in horses and, commonly, in humans [28,29]. Horses might acquire livestock-associated MRSA, which, at least in Western Europe, is usually represented by CC398. CC130 might also be livestock-associated, as it is common among small ruminants [30,31,32]. For some lineages, such as CC816 and CC8115, not much is known; they might have been transmitted from other animals to horses, or vice versa [33].

Finally, there are lineages that might be considered naturally adapted to horses. These include CC1 and CC1660 (also known as CC350) which have already been observed in Switzerland, Germany, Austria and other countries [34,35,36]. This raises the question of which virulence factors or other genetic determinants are associated with the ability to infect or to colonize horses. These lineages also might be observed in humans and there are several ubiquitous, near-pandemic CC1 strains, methicillin-susceptible *S. aureus* (MSSA), and MRSA in humans [37,38,39] as well as in livestock [40]. Therefore, it would be interesting to study differences between human and equine CC1 strains to check whether strains from humans can colonize and/or infect horses or vice versa, and to see which genes might pre-determine host specificity.

*S. aureus* isolates of CC1 and CC1660 have been detected in the Robert Koch Institute’s isolate collection obtained from human and equine samples, respectively. The detection of these equine-associated CCs in human samples is notable and warrants monitoring since a zoonotic spillover of such CCs might be a remarkable aspect for public health in the One Health context. Therefore, the aim of this study was to investigate the genetic relationships of these isolates using whole-genome sequencing (WGS) and bioinformatic analyses, as well as their virulence and resistance properties.

## 2. Results and Discussion

### 2.1. Genetic Relationships

In total, 75 isolates were confirmed as CC1, including five cases with two different phenotypes from the same sample (Figure S1), and 16 isolates as CC1660, based on the seven MLST alleles. Among the CC1 isolates, nine STs were detected, 61 isolates belonged to ST1, four to ST81, two to ST9569 and single isolates belonged to ST9300, ST9570, ST9571, ST9572 and ST9573, with the latter four being new STs identified during this study. Moreover, three isolates (18-02051-36, 18-02052-37, 18-02054-39), also assigned to CC1 as single-locus variants, were identified, in which a deletion of 285 bp within the *arcC* gene had occurred. Because the 285 bp deletion includes the region used to define the *arcC* allele in MLST, no ST could be assigned for these isolates. Among the CC1660 isolates, four STs were detected, namely ST1660 present in 13 isolates, as well as ST9574, ST9575 and one with a different *arcC* deletion in single isolates. It should be noted that the novel ST also has a single bp deletion in the homopolymeric region between nucleotides 594 and 605 within the *arcC* gene. This deletion resulted in a frame shift which produced an early stop codon at amino acid position 211 (see also Section 2.2).

**Figure 1:**
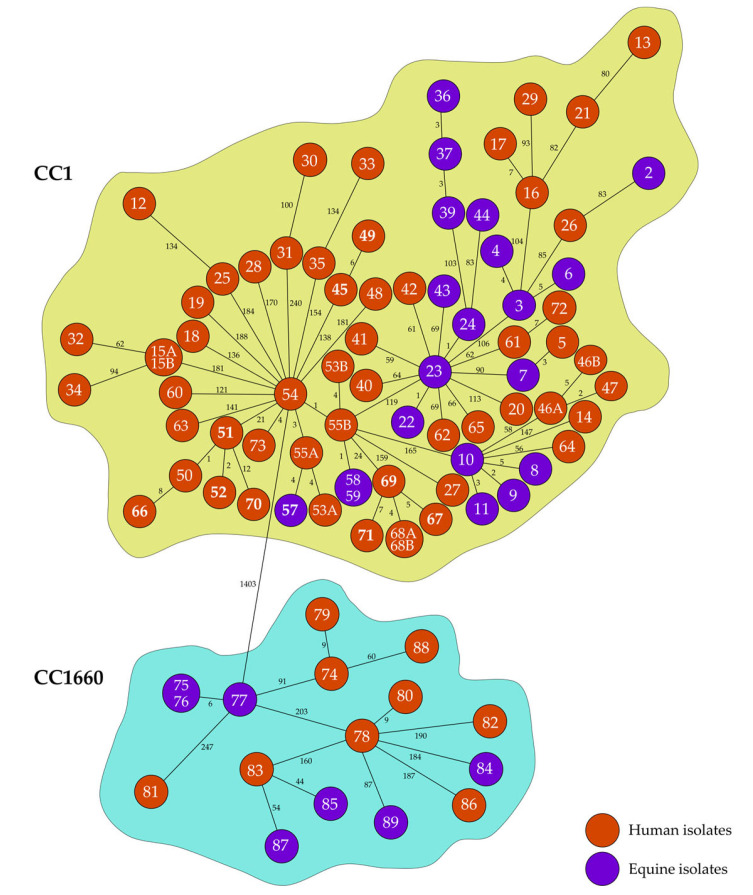
Minimum spanning tree based on the cgMLST allelic profile of the 91 *S. aureus* isolates (1492 columns; 1861 columns for distance calculation, 369 columns with missing values with at least one sample removed). The distance between isolates is indicated by the number next to the line (length of line not proportionate). The correlations of numbers and isolate IDs are given in Table S1. The distance information of the single isolates is displayed as a heatmap in Table S2.

In comparison to the conventional MLST with only seven target genes (https://pubmlst.org/; accessed on 22 October 2025), cgMLST targets a fixed set of genome-wide distributed core genes, including about 400 to over 4000 alleles (https://www.cgmlst.org/ncs; accessed on 22 October 2025), for different bacterial species, including 1861 alleles for *S. aureus* (https://www.ridom.de/seqsphere/cgmlst/; accessed on 22 October 2025) [41]. The cgMLST revealed a minimum difference between the two CCs of 1398/1492 alleles and clearly separated the two CCs (Table S2). The difference between the isolates ranged from 0 to 288 alleles in CC1 and from 0 to 249 alleles in CC1660 (Figure 1, Table S2).

Because multiple samples were collected from nine equine clinics (ECs), we compared the cgMLST results, sample metadata, and geographic origin (Table S1, Figures S2 and S3) to assess isolate relatedness within and between clinics. Related isolates could be detected in six of the nine equine clinics. The three equine isolates of each of the clinics EC6 and EC7 (including two isolates from the same sample) were classified as related, using the classification proposed by Cunningham et al. [42]. Fourteen isolates from clinic EC1 were investigated, including 12 CC1 isolates (nine human, with two samples containing paired isolates, and three equine). All CC1 isolates were classified as possibly related, with some subsets (nine and three isolates) showing close relatedness. Within the ten CC1 isolates (one human, nine equine) from EC2 as well as the six human CC1 isolates, including two isolates from the same sample, from EC4, all but one of the isolates per equine clinic were closely related. Two of four human isolates in EC5 were also related. In contrast, the seven isolates from EC3 as well as the two isolates from EC8 and EC9 were not related. The detection of equine-related *S. aureus* isolates of these CCs among humans in equine clinics could be explained as simple contamination due to close contact with horses. Since no repeated testing was performed as in previous studies, e.g., on CC398 [43], a differentiation between contamination, transient or permanent colonization of the CCs among humans cannot be made. Therefore, further investigation of the presence and carriage of *S. aureus* CC1 and CC1660 among horses would be interesting to monitor a possible spillover of these equine-related CCs to humans and elucidate a potential zoonotic risk of these isolates.

The *spa* typing revealed ten *spa* types among the CC1 isolates comprising t114 (*n* = 1), t127 (*n* = 62), t273 (*n* = 1), t559 (*n* = 2), t922 (*n* = 1), t1383 (*n* = 2), t1491 (*n* = 1), t1508 (*n* = 3), t1405 (*n* = 1), and t18599 (*n* = 1), with t127 being the most prevalent, which is in accordance with the data from the ridom database https://spa.ridom.de/spa-t127.shtml (accessed on 22 October 2025). All *spa* types were related, but differed in the number of repeats, ranging from four repeats (t18599) to eleven repeats (t1491), which can mainly be explained by one or two deletion or insertion events in the variable region of the *spa* gene. Moreover, single point mutations in three different positions, compared to the most common alleles, were observed (Figure S4a).

Among the CC1660 isolates, five *spa* types were detected, comprising four isolates with t549, seven isolates with t3043, three isolates with t11926, as well as single isolates with t12047 and t15977. The five *spa* types detected among the CC1660 isolates comprised five to twelve repeats. All of the detected *spa* types were related and differed in the number of repeats and or exchanges of repeats, which can be explained as point mutations in two different positions (Figure S4b).

### 2.2. Deletions Within the arcC Gene

**Figure 2:**
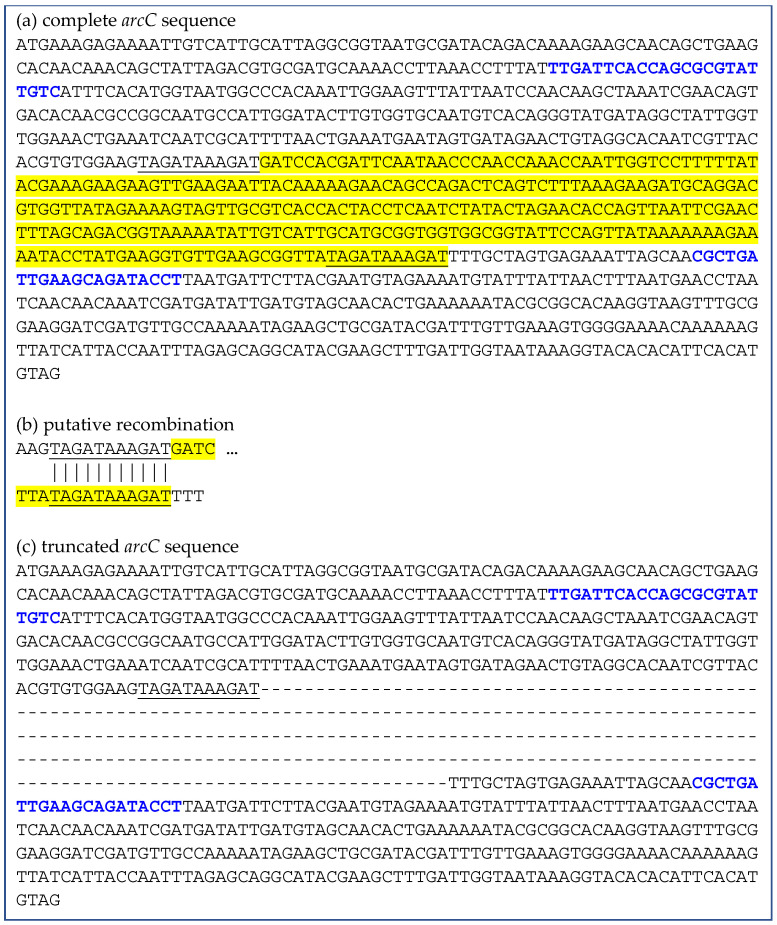
Illegitimate recombination resulting in a 285 bp deletion within the *arcC* sequence. (**a**) Complete *arcC* sequence, (**b**) Putative recombination event and (**c**) Truncated *arcC* sequence. The recombination sites are underlined and the deleted area is indicated in yellow. The primer binding sites within the *arcC* are displayed in blue. |= identity of bases in the recombination site (**b**); – = deleted base (**c**).

A 285 bp deletion (corresponding to 95 amino acids, namely the amino acids at positions 117–211) was identified in the *arcC* gene (Figure 2), which—if complete—encodes a 313-amino acids carbamate kinase, during whole-genome sequencing of three isolates (18-02051-36, 18-02052-37, 18-02054-39). The deletion of amino acids 117–211 is located between the primer binding sites (Figure 2) and results in a change in the PCR product size from 570 bp to 285 bp (Figure 2 and Figure S5). This finding was confirmed via PCR amplification and sequencing of the PCR products (Figure S5). Sequence analysis suggested that the deletion likely resulted from an illegitimate recombination event (Figure 2).

As shown by Ramón-Maiques et al. [44], the positions N51, K128, K209, and D210 play a role in the ternary complex active-site architecture and the catalytic mechanism proposed. These positions correspond to the positions N51, K128, K213, and D214 in the *S. aureus* ArcC carbamate kinase (GenBank: BAB58794.1; ABD21772.1). Ramón-Maiques et al. [44] observed an extreme negative effect of a mutation in position 128 (K128A) pointing towards a key role of the protruding subdomain, hanging over the carbamate kinase active center, as an exclusive and characteristic carbamate kinase feature for substrate binding and catalysis. A mechanism connecting carbamoyl phosphate site occupation with the protruding subdomain approach, involving V206-I207 (positions V210-I211 in *S. aureus*) in the carbamoyl phosphate site and P162-S163 (positions A164-S165 in *S. aureus*) in the protruding subdomain stem, was also identified [44]. From the important amino acid positions described by Ramón-Maiques et al. [44], the respective correlating positions K128, V210-I211 and P162-S163 are part of the deleted region in the three *S. aureus* isolates of the present study. Since the positions K213 and D214 are involved in the putative recombination event, it cannot be clarified whether this position is part of the deletion, and if so, whether the homologous part can take over its function (Figure 2). Therefore, it can be assumed that the deletion detected within the *arcC* gene results in the loss of function of the ArcC protein, inhibiting the final step of the microbial fermentative catabolism of arginine, agmatine, and oxalurate/allantoin [44].

The delayed and incomplete reaction of the anaerobic arginine dehydrolase (Figure S6) might be explained by the presence of an additional carbamate kinase gene, which showed 99.9% identity at the nucleotide sequence level and 100% at the amino acid level [GenBank: AFD28597.1] to an *arcC* gene as indicated in locus 2 [GenBank: JQ031649.1] by Zhang et al. [45]. This gene showed only 47.3% homology at the amino acid level compared to the *arcC* gene used in the *S. aureus* MLST scheme indicated as locus 1 (GenBank: NC_007793.1 [2,779,133–2,778,192]) by Zhang et al. [45]. This additional carbamate kinase can possibly cover, at least partly, for the function of the truncated carbamate kinase ArcC as supported by the results from the anaerobic arginine dehydrolase testing. It should be noted that the arginine catabolic mobile element (ACME), described to harbor a third carbamate kinase [45], is not present in the investigated *S. aureus* collection.

The human CC1660 isolate 13-03859-1660.7 also carried an adenine nucleotide deletion in the *arcC* gene within the homopolymeric region (594–605). This results in changes in the protein (difference of 10 amino acids, positions 201 to 210) as well as an earlier stop codon at position 211. The newly described deleted *arcC* is designated as “allele 613” in the locus SAUR2853 (=*arcC*) at pubmlst.org (accessed on 22 October 2025). This deletion resulted in a delayed reaction during anaerobic arginine dehydrolase testing, which, however, did not seem as impacted as the one observed for the isolates with the 95 amino acids deletion (Figure S6).

### 2.3. Antimicrobial Resistance

#### 2.3.1. Antimicrobial Susceptibility Testing and Interpretation of the Results

Antimicrobial susceptibility testing was performed for 31 antimicrobial agents, and the respective MIC distributions of all isolates are displayed in Table 1. For the interpretation of the susceptibility testing results for penicillin, different breakpoints for human and equine isolates were available and classified 16 human isolates (10 CC1, 6 CC1660) with MICs of ≤0.12 µg/mL and three equine isolates (all CC1660) with MICs of ≤1 µg/mL as susceptible. The remaining 48 human (45 CC1, 3 CC1660) and 24 equine isolates (20 CC1, 4 CC1660) were classified as resistant. For horses, clinical breakpoints for ampicillin are also available, classifying all but three isolates (all CC1660) as resistant, which is in accordance with the results from the penicillin testing. For oxacillin, the human-specific clinical breakpoints were used for all isolates, since no veterinary-specific clinical breakpoints are available. Three human and five equine CC1 as well as two equine CC1660 are classified as oxacillin-resistant, which are, therefore, considered as MRSA (see also Section 2.3.2).

Tetracycline resistance was detected among 18 human and ten equine CC1 isolates as well as single human and equine CC1660 isolates. For doxycycline, all but one of the human CC1 isolates were classified as doxycycline-susceptible with the remaining one being classified as intermediate. Among the equine isolates, 12 (eight CC1, four CC1660) were classified as susceptible, four (two of each CC) as intermediate, and ten CC1 isolates as well as the remaining CC1660 isolate as resistant to doxycycline based on the horse-specific clinical breakpoints. It should be noted that there was a good correlation between tetracycline and doxycycline resistance among the equine isolates. However, this correlation could not be observed for the human isolates when using only the breakpoint-based classification. Still having a closer look at the MIC distributions, it can be seen that all but one of the human tetracycline-resistant isolates had elevated doxycycline MICs of 2 µg/mL to 4 µg/mL and were classified as doxycycline-susceptible, whereas the remaining isolate (MIC: 8 µg/mL) was classified as intermediate to tetracycline.

Aminoglycoside resistance was also commonly seen in this collection, with 31 isolates, including 18 human (17 CC1, one CC1660) and 13 equine (ten CC1, three CC1660) isolates being gentamicin-resistant. A single equine CC1660 isolate was classified as intermediate. Moreover, elevated MICs ≥ 4 µg/mL for neomycin and ≥128 µg/mL for streptomycin were detected among 30 and 11 isolates, respectively.

Erythromycin resistance was detected only in nine CC1 isolates (five human, four equine). The observation that all isolates had comparably low clindamycin MICs of ≤2 µg/mL might point towards inducible macrolide resistance [46] (see Section 2.3.3). Resistance to the combination sulfamethoxazole/trimethoprim was detected in 19 isolates based on MICs of ≥4/76 µg/mL, comprising 16 human CC1 and three equine CC1 isolates.

All human isolates were classified as ciprofloxacin-susceptible with MICs of ≤1 µg/mL. In comparison, all but three isolates of the equine collection were classified as susceptible based on enrofloxacin MICs of ≤0.12 µg/mL. The remaining three CC1 isolates were classified as resistant by their MIC of 0.5 µg/mL.

A single human CC1 isolate (10-00442-18) had an elevated florfenicol MIC of 64 µg/mL. All isolates were susceptible to the oxazolidinone linezolid, the glycopeptide vancomycin, the streptogramin combination quinupristin/dalfopristin and the pleuromutilin tiamulin. This is a rather favorable situation, but it should be noted that the Antimicrobial Advice ad hoc Expert Group (AMEG) classified glycopeptides, oxazolidinones and streptogramins in Category A (“Avoid”) of the European Medicines Agency (EMA), which are, therefore, not authorized in veterinary medicine and restricted to use in human medicine in the EU [47].

#### 2.3.2. Correlation of Phenotypic and Genotypic Resistance Properties

Antimicrobial susceptibility testing revealed 18 distinct phenotypic resistance patterns among the isolates, whereas whole-genome sequence analysis identified 19 genotypic resistance patterns (Table 2). Among the 91 *S. aureus* isolates, 18 were susceptible to all antimicrobial agents tested and 31 isolates were only resistant to penicillin. One penicillin-resistant isolate showed additional phenotypic resistance to oxacillin only. Nine additional isolates were phenotypically methicillin-resistant. Methicillin resistance is commonly mediated by the genes *mecA* and *mecC* [48,49]. This includes all β-lactams licensed for veterinary use. The β-lactams, which are effective against MRSA, such as the cephalosporin ceftobiprole [50], are restricted for use in humans by the AMEG category A [47]. In the present collection, only seven methicillin-resistant isolates carried the *mecA* gene (see also Section 2.3.3). In total, three equine isolates were phenotypically oxacillin-resistant, with a low oxacillin MIC of 4 µg/mL (12-00973-22 [CC1]; 12-00626-1660.2 and 12-00971-1660.3 [CC1660]), but negative for the respective resistance genes *mecA* and *mecC*. The finding of phenotypic methicillin resistance with low MICs without a known methicillin resistance gene is described as borderline oxacillin-resistant *S. aureus* (BORSA). A possible reason for the observation of phenotypic methicillin resistance without the presence of a *mec* gene or mutations in the genes for penicillin-binding proteins could be due to an overexpression of the *blaZ* gene as described previously [51]. However, the comparison of the *blaZ* operons (*blaZ*, *blaI*, *blaR*) in the present study revealed that the same nucleotide sequence variants were not only present in the methicillin-resistant but also in methicillin-susceptible isolates.

Tetracycline resistance was detected in 30 isolates, among which 22 harbored the gene *tet*(L), whereas the remaining eight carried a *tet*(K) gene. This finding also corresponds well with the study by Scholtzek et al. with all three tetracycline-resistant isolates being positive for *tet*(L) [51], which is also the most common tetracycline resistance gene in our collection. In comparison to other studies on *S. aureus* that describe the presence of two or more tetracycline resistance genes in the same isolate [52,53], only one tetracycline resistance gene was detected per isolate.

Resistance to gentamicin was present in 31 isolates and it was in all isolates mediated by the *aacA-aphD* gene (also known as *aac(6*′*)-aph(2*″*)*). The single gentamicin-intermediate isolate was also positive for *aacA-aphD*. This is in accordance with previous studies with BORSA isolates of CC1 and CC1660, where all of these isolates showed *aacA-aphD*-mediated gentamicin resistance [34,51]. Gentamicin resistance is also common among MRSA ST8 isolates isolated from horses in the USA [28]. Neomycin resistance was also very common in this collection, being present in 30 isolates, 22 of which carried the *aadD* gene (also known as *ant(4*′*)-Ia*), whereas the remaining eight carried *aphA3*. The eight *aad*(E) (also known as *ant(6)-Ia*)-positive and the three *str*-positive isolates had streptomycin MICs of ≥128 µg/mL.

In total, 19 isolates proved to be phenotypically resistant to the combination trimethoprim/sulfamethoxazole, which was in agreement with the presence of *dfrS1* and/or *dfrG* (mediating trimethoprim resistance) as well as the mutations F17L and A184V in the *folP* gene (mediating sulfonamide resistance). However, an additional 15 isolates harbored a *dfr* gene (*dfrS1*, also known as *dfrA* [*n* = 10], *dfrK* [*n* = 4], *dfrG* [*n* = 1]) and two had the F17L mutation in the *folP* gene, but were classified as trimethoprim/sulfamethoxazole-susceptible by broth microdilution. This finding is in accordance with previous studies showing that resistance mechanisms for both components, trimethoprim (*dfr* genes) and sulfonamides (*folP* gene mutations), are needed to confer a trimethoprim/sulfamethoxazole resistance phenotype [54]. The finding that *dfrS1* is the most common trimethoprim resistance gene is in accordance with the findings of Sieber et al. [34] who detected this gene in all 18 CC1 and CC1660 BORSA isolates tested. Among the resistant isolates, at least one *dfr* gene is present in combination with two mutations (F17L and A184V) within the *folP* gene (Table 2).

Nine isolates were resistant to erythromycin mediated by the *erm*(C) gene (conferring resistance to macrolide, lincosamide, and streptogramin B antibiotics), but showed unimodal distributions for the other macrolides and lincosamides tested (Table 1). This points towards inducible macrolide resistance [46]. As described previously [46], the regulatory region upstream of the *erm*(C) gene was complete and showed no mutations. This finding is important for the choice of the antimicrobial agents, since *erm* genes are only induced by 14- and 15-membered macrolides, such as erythromycin and azithromycin, but not by 16-membered macrolides (e.g., tylosin), lincosamides (e.g., clindamycin) and streptogramin B antibiotics [46]. In the presence of inducibly expressed *erm* genes, the aforementioned non-inducers should not be used for therapeutic purposes, since mutations in the regulator region can occur rapidly and result in constitutive *erm*(C) expression [46].

Fluoroquinolone resistance is known to be mediated by mutations in the quinolone resistance-determining regions (QRDRs) of the genes for DNA topoisomerase IV (*grlA* and *grlB*) and DNA gyrase (*gyrA*, *gyrB*). In three equine and 12 human CC1 isolates, the GrlA amino acid replacement S80Y has been detected. This corresponds well with the classification of equine isolates as resistant. In contrast, the human isolates with the same GrlA amino acid replacement were classified as susceptible. Having a closer look at the MIC values, it can be noted that these human isolates also had elevated MIC values, corresponding well with the MIC values of the equine isolates. Therefore, the different clinical breakpoints used for the human and equine isolates resulted in a different categorization despite similar MIC values and the presence of a mutation in the QRDR (see also Section 2.3.1; Table 2).

A single isolate was classified as phenicol-resistant and carried the *fexA* gene mediating resistance to non-fluorinated and fluorinated phenicols [55].

#### 2.3.3. SCC Elements

Seven CC1 isolates were identified as MRSA. All harbored SCC*mec* IVa (2B) elements. For the seven *mecA*-positive isolates, *dru* typing—a recognized MRSA subtyping method targeting a variable-number tandem repeat region located within the SCC*mec* element between *mecA* and IS*431*—was performed [56]. Four isolates harbored the *dru* type dt9y (5a-2d-4a-0-2d-5b-3a-3b-4e), whereas the remaining ones represented *dru* type dt10a (5a-2d-4a-0-2d-5b-3a-2g-3b-4e). Actually, the detected *dru* types are related and dt9y lacks repeat 2g, which is the eighth repeat in dt10a.

Of note, in all seven CC1-MRSA-IVa sequences, genes *ydil2-*SCC and C5QAP8 (locus tags KOJLHLHF_00002 and _00004 in GenBank MT380478.1) were identified as well as *npd = pnoA*-SCC (KOJLHLHF_00006) in five out of the seven isolates. The presence of these genes corresponds to a large insertion directly downstream of *orfX* previously described to cause false-negative results in some commercial assays for molecular MRSA screening [57].

In addition, two CC1 isolates carried an SCC element without *mecA* or *mecC* genes, but comprising of the fusidic acid resistance gene *fusC*, the *tirS* gene for a staphylococcal Toll-like/interleukin-1 receptor and recombinase genes *ccrA1/B1*. The element comprising these genes is already known to occur in CC1 strains, corresponding, for instance, to locus tags SAS0043, SAS0038, SAS0033 and SAS0032, respectively, in MSSA476 (GenBank BX571857.1).

### 2.4. Clonal Complex-Specific Virulence Factors

All CC1 isolates (*n* = 75) harbored *agr* group III and capsule type 8 alleles. The *lukF/lukS/*hemolysin gamma locus (corresponding to locus tags MW2342 to MW2344 in the CC1 reference sequence of MW2, GenBank BA000033.2) and the leukocidin D/E genes (MW1767/1768) were always present. Another leukocidin locus, *lukA/B* (MW1941/1942), was detected in 73 out of 75 (97.3%) isolates. The enterotoxin H gene (*seh*; MW0051), located on a genomic island downstream of *orfX* and the SCC*mec* integration site, was identified in 74 (98.7%) isolates. Two genes encoding adhesion factors known to correlate with CC affiliations were also examined. These were the *sasG* gene (MW2416), encoding the *S. aureus* surface protein G, and *cna* (MW2612), encoding a collagen adhesion protein, which were present in 74 (98.7%) and 70 (93.3%) isolates, respectively.

The combination of *agr* group III, capsule type 8, *seh*, *sasG* and *cna* is characteristic for CC1. *Agr* group III and capsule type 8 can also be found in isolates of CC93, CC30 (which also carried the *cna* gene), CC80 and CC88 (which also carried the *sasG* gene), CC509/ST 207 as well as ST154 (which also carried the *cna* and *sasG* genes) isolates. The *seh* gene is rarely—if at all—found outside of CC1 isolates [37], whereas *lukA/B* genes can be found in virtually all *S. aureus* isolates [33]. All CC1660 isolates (*n* = 16) carried *agr* group II alleles and capsule type 5 genes. Genes of the *lukF/lukS*/hemolysin gamma and the *lukA/B* and *lukD/E* loci were universally present. Isolates of this clonal complex lacked the *seh* gene, but they always carried the enterotoxin gene cluster (*egc*, comprising *selg*, *seli*, *selm*, *sen*, *selo*, *selu*), although the *selg* gene remained undetected as well as *seli* and *selm* in one isolate. The absence of *selg* appears to be a general property of CC1660 as shown by array experiments with additional, epidemiologically unrelated isolates (Monecke, personal communication). All isolates harbored an additional enterotoxin-like gene (“*selu*2”, locus tag UC18_08930 from CP010526.1). The *cna* gene was present whereas *sasG* was absent in all CC1660 sequences.

The combination of *agr* group II and capsule type 5 alleles was seen in all isolates of CC5 (which also carried the *egc* and *sasG* genes, but lacked *cna*), CC9 (which also harbored only *egc*), as well as ST573 and ST1772 (which also carried the *egc*, *sasG* and *cna* genes) [37].

### 2.5. Virulence Factors on Mobile Genetic Elements

Virulence factors *lukP/lukQ* (=*lukP*Q leukocidin, horse-specific), *sak* (staphylokinase), *sak*_phi-42e (putative horse-associated kinase), *scn* (staphylococcal complement inhibitor), *scn2* (complement inhibitor paralog from a staphylococcal pathogenicity island (SaPI), locus tag SAPIG0482 from AM990992.1), *scn*-eq (putative horse-associated complement inhibitor), enterotoxin genes *sea*, *seb*, *sec*, *sek*, *sel*, *seq*, the toxic shock syndrome toxin gene *tst-1* and *vwb3* (a SaPI-borne gene encoding a “van Willebrand factor”-binding protein, locus tag SAPIG0483 from AM990992.1) were present in various combinations. The distribution and co-occurrence of the aforementioned virulence genes in CC1 and CC1660 isolates from humans and horses are summarized in Table 3.

Horse-specific leukocidin genes *lukP/luk*Q as well as *scn-eq* genes are located on prophages that integrate into the chromosomal *lip2* (=*geh*) gene [33]. The respective prophages differ with respect to their structures and gene contents into three groups, of which one was seen in CC1 and CC97, another one was detected mostly in CC350, but also in CC1, CC97, CC133 and CC398, whereas a third one was solely found in CC816 and CC8115 [33]. The enterotoxin gene *sea* has been identified on β-hemolysin-converting phages in isolates of CC5 and CC8, but occasionally also in CC1, CC6, CC15, CC30, CC59, CC398, ST239, ST426, and ST772. The enterotoxin B gene *seb* has so far also been detected in CC361 and together with the genes *sek* and *seq* in isolates of CC8 and CC59; *sek* and *seq* have also been seen in CC45, CC398, ST239 and ST426 [37]. The gene *tst-1* for toxic shock syndrome toxin 1 also occurs in isolates of CC5, CC8, CC22, CC30, CC45, CC361, ST426, CC705 and ST834 [37].

Phage-borne leukocidin genes *lukF-PV/lukS-PV* (Panton–Valentine leukocidin), plasmid-borne enterotoxins (*sed*, *sej*, *ser*), the gene encoding a chemotaxis-inhibiting protein (*chp*), exfoliative toxins (*etA*, *etB*, *etD*, *etE*) and epidermal cell differentiation inhibitor genes (*edinA*, *edinB*, *edinD*) were not detected.

## 3. Materials and Methods

### 3.1. Isolate Collection

The study is based on a set of 91 *S. aureus* isolates belonging to CC1 (*n* = 75) and CC1660 (*n* = 16) of human and equine origin obtained within the period from 2006 to 2022 by the Robert-Koch-Institute, Wernigerode, Saxony-Anhalt, Germany, during previous studies, and some of the isolates have already been published previously [26,58]. The collection consists of isolates from humans (employees) and horses obtained from a study in equine clinics and from humans and horses in the community in Germany. It includes 75 CC1 and 16 CC1660 isolates. Among the 75 CC1 isolates, 55 were of human origin and 20 of equine origin. From five human samples, two phenotypically distinct isolates each were analyzed (Figure S1). Among the 16 CC1660 isolates, nine were of human and seven of equine origin. The geographic origin is only given on a federal state level for data protection reasons (Table S1, Figure S2). In eight cases, more than one sample of human and/or equine origin from the same equine clinics was investigated. In one case, three samples from the same horse (12-00973-22, 12-00974-23, 12-00975-24) were provided from a veterinary diagnostic laboratory and included.

### 3.2. Whole-Genome Sequencing (WGS) and Sequence Analysis

#### 3.2.1. Whole-Genome Sequencing

All isolates were subjected to WGS. Whole-cell DNA extraction was performed using the QIAamp® DNA Mini Kit (QIAGEN, Hilden, Germany) with some adaptations for staphylococci as described previously [51]. Initially, staphylococcal cells were mixed with 25 µL lysostaphin solution (0.1 mg/mL) followed by incubation at 37 °C for 25 min. Subsequently, 25 µL proteinase K (0.1 mg/L) and 75 µL TE buffer were added and the mixture was incubated at 37 °C for another 25 min. Finally, 2 µL RNAse A (2 µg/µL) and 75 µL PBS were added and mixed. Thereafter, the protocol for the kit was followed starting with the addition of AL buffer [51]. The libraries were prepared using the Nextera XT library preparation kit (Illumina Inc., San Diego, CA, USA) according to the manufacturer’s instructions. The 2 × 300 bp paired-end sequencing in 40-fold multiplexes was performed on the Illumina MiSeq platform. Trim Galore v0.6.10 (RRID: SCR_011847) and FastQC v0.12.1 (https://www.bioinformatics.babraham.ac.uk/projects/fastqc/; accessed on 22 October 2025) were used for adapter trimming and quality checking. The genome sequences were de novo assembled using Unicycler v0.4.9 [59] and SPAdes v3.15.5 [60].

#### 3.2.2. Molecular Typing and Phylogenetic Analysis

Conventional MLST for *S. aureus* is based on seven housekeeping genes (*arcC* [carbamate kinase], *aroE* [shikimate dehydrogenase], *glpF* [glycerol kinase], *gmk* [guanylate kinase], *pta* [phosphate acetyltransferase], *tpi* [triosephosphate isomerase], and *yqiL* [acetyl coenzyme A acetyltransferase]) for *S. aureus* [5]. The respective alleles were deduced from the whole-genome sequences using the software program mlst from the PubMLST database [61]. For the sake of convenience, STs were then clustered in CCs. There are slightly different definitions of a CC, e.g., isolates with five of the seven alleles in common [6,7] or isolates that “match the central genotype (ST) at four or more loci unless they more closely match another central genotype” (https://pubmlst.org/organisms/staphylococcus-aureus/clonal-complexes; accessed on 22 October 2025). In our case, all isolates were single-locus variants of the central genotype, which is in accordance with both definitions. It should be noted that the CC assignment commonly concurs with the *agr* group, the capsule type affiliations and the carriage of markers on major genomic islands (*seh*, ORF CM14, *egc* locus, *ssl* locus) [37].

In addition, *spa* typing and SCC*mec* typing were extracted from the WGS sequences using the software programs spaTyper [62] and SCCmecFinder v1.2 (https://cge.food.dtu.dk/services/SCCmecFinder/; accessed on 22 October 2025), respectively. The *dru* types were identified according to the drutyping.org database [56] using the basic local alignment search tool (BLAST) function in Geneious v11.1.5 (Biomatters, Ltd., Auckland, New Zealand).

The 91 genomes were also subjected to phylogenetic analysis with chewBBACA v3.3.10 [63] using the *S. aureus* core genome MLST (cgMLST) approach [41]. A minimum spanning tree was built based on a distance matrix of the core genome allelic profiles to illustrate the clonal relationships between the isolates. Overall, 1492 of 1861 potential target genes were included in the analysis by removing 369 columns that were missing in at least one sample. GrapeTree [64] was used to visualize the minimum spanning tree.

PCR amplification of the *arcC* gene was performed for three isolates with a 285 bp deletion in the *arcC* gene, an isolate with a single bp deletion as well as an isolate with a complete *arcC* gene, using the primers and the protocol described previously for MLST by Enright et al. [5] (Table S3) and subsequent agarose gel electrophoresis before Sanger sequencing at LGC Genomics GmbH (Berlin, Germany) for confirmatory reasons.

### 3.3. Anaerobic Arginine Dehydrogenase Testing

The testing of the anaerobic use of arginine was performed for selected isolates using DIATABS™ ARGININE DIHYDROLASE (ADH) (Rosco Diagnostica A/S, Albertslund, Denmark) according to the manufacturer’s instructions (https://www.keyscientific.com/files/Other%20Manufacturers/Rosco/DiaTabs/Rosco%20DiaTabs.pdf; accessed on 22 October 2025). Bacterial suspensions of McFarland 4 were prepared in saline. One ADH Diagnostic tablet was added to 0.25 mL bacterial suspension and anaerobic conditions were generated by adding sterile paraffin oil. The results were read after 4 h and 24 h of incubation at 37 °C.

### 3.4. Antimicrobial Susceptibility Testing

Antimicrobial susceptibility testing was performed by broth microdilution according to CLSI standards. Tests were usually performed once, but were repeated if quality control (QC) was out of range or if results were inconclusive. The McFarland 0.5 is adjusted using the direct colony suspension method: 60 µL of this bacterial suspension is added to 12 mL cation-adjusted Mueller–Hinton broth and incubated for 16–20 h or 24 h (for oxacillin and vancomycin only) in ambient air. Custom-made microtiter plates (MSC diagnostics, Swalmen, The Netherlands) containing 30 antimicrobial agents [48,49,65] were used, which are also used in the German National Resistance Monitoring program GE*RM*-Vet [66]. The test panel included β-lactams (oxacillin, penicillin, ampicillin, amoxicillin/clavulanic acid, imipenem, ceftiofur, cefquinome, cefalothin, cefotaxime, cefoperazone), macrolides (erythromycin, tylosin tartrate, tulathromycin, tilmicosin), lincosamides (clindamycin, pirlimycin), quinolones (ciprofloxacin, enrofloxacin, marbofloxacin), aminoglycosides (gentamicin, streptomycin, neomycin), tetracyclines (tetracycline, doxycycline), a phenicol (florfenicol), a folate pathway inhibitor combination (sulfamethoxazole/trimethoprim), a pleuromutilin (tiamulin), a glycopeptide (vancomycin), a streptogramin (quinupristin/dalfopristin) and an oxazolidinone (linezolid). *S. aureus* ATCC^®^ 29213 served as the QC strain. Since the collection contains human and equine isolates, species-specific clinical breakpoints were applied as far as possible using the clinical breakpoints from CLSI document M100 for the human isolates [49] and from the CLSI document VET01S for the equine isolates [48]. For our collection, human-specific clinical breakpoints for 12 antimicrobial agents were available, namely penicillin, oxacillin, erythromycin, clindamycin, ciprofloxacin, gentamicin, tetracycline, doxycycline, trimethoprim/sulfamethoxazole, linezolid, vancomycin and quinupristin/dalfopristin [49]. For the equine isolates, equine-specific clinical breakpoints for penicillin, ampicillin, enrofloxacin, and doxycycline as well as human-adopted clinical breakpoints for oxacillin, erythromycin, gentamicin, tetracycline, trimethoprim/sulfamethoxazole, linezolid and vancomycin were applied [48]. Thus, in some cases, the human clinical breakpoints were adopted for veterinary isolates; however, these results should be interpreted with caution due to species-specific differences in dosage and pharmacokinetics/pharmacodynamics. As a consequence of the different species-specific breakpoints for certain compounds, any discussion on resistance properties and phenotypic effects of resistance genes must focus on raw MIC values rather than on mere susceptible/intermediate/resistant classifications, especially if it deals with pathogens affecting multiple host species.

### 3.5. Detection of Resistance Genes and Resistance-Mediating Mutations

Known antimicrobial resistance genes were identified with ABRicate v1.0.1 (https://github.com/tseemann/abricate; accessed on 22 October 2025) using the NCBI AMRfinder [67] and the CGE ResFinder database [68]. The assembled WGS sequences were used as fasta files for analysis using the default settings. Chromosomal point mutations conferring AMR were detected by applying PointFinder [69] as part of the ResFinder tool [68] using default settings. All results were confirmed with Geneious v11.1.5 (Biomatters, Ltd., Auckland, New Zealand), a bioinformatic tool for sequence analysis, BLAST [70], a Basic Local Alignment Search Tool to find regions of similarity between biological sequences, and the UniProt Knowledgebase [71] to identify protein sequences and their function.

### 3.6. Determination of Virulence Properties

Virulence genes were identified with ABRicate v1.0.1 (https://github.com/tseemann/abricate; accessed on 22 October 2025) using the VFDB database [72]. As described for the determination of antimicrobial resistance genes, the assembled WGS sequences were used as fasta files for analysis using the default settings. All results were confirmed with Geneious v11.1.5 (Biomatters, Ltd., Auckland, New Zealand), BLAST + 2.17.0 [70], and the UniProt Knowledgebase [71]. In addition, sequences of the probes of previously published DNA microarrays [37,73,74] were mapped against the genome sequences of the isolates.

## 4. Conclusions

In summary, comprehensive genetic and phenotypic characterization of *S. aureus* isolates from humans and horses revealed a clear separation between the 75 CC1 and the 16 CC1660 strains. No human- or horse-associated subpopulations within the CCs could be observed. Notable alterations were found in the *arcC* gene of four isolates with potential functional consequences. Isolates within each clonal complex carried related *spa* types, supporting the established epidemiological linkage.

A total of 18 phenotypic and 19 genotypic antimicrobial resistance patterns were identified, including numerous isolates that were resistant to more than three classes of antimicrobial agents. The application of species-specific clinical breakpoints for different fluoroquinolones used in human or veterinary medicine showed that isolates with similar MICs and the same *grlA* mutation may be classified either as susceptible or resistant. This observation points towards a problem in resistance surveillance studies when using species-specific clinical breakpoints rather than epidemiological cut-off values or raw, uninterpreted MIC values. The detection of diverse mobile genetic elements, such as SCC*mec* cassettes and phages, highlights the dynamic potential for horizontal gene transfer of virulence and antimicrobial resistance determinants. We also observed that equine-adapted strains carried genes such as *lukP/lukQ*, *sak*_phi-42e or *scn*-eq [33] and that human as well as human-adapted strains, carrying prophages with immune-evasion cluster genes in various combinations (*scn*, *sak*, *chp*, *sea*) [75,76], were present in horses. Therefore, it would be interesting to investigate whether these strains adapted to a specific host can stably colonize other host species, and whether species-specific virulence factors, such as the human toxic shock syndrome toxin or the horse-specific leukocidin, are also functional in other hosts. For this, a detailed characterization of the respective strains in terms of surveillance of zoonotic transmission—as provided in this study—is indispensable. The findings of this study underscore the importance of ongoing surveillance and molecular monitoring of equine-associated *S. aureus* clonal complexes—not only in horses and humans, but also in animals with close contact to these hosts—to better understand and mitigate potential risks to animal and public health in a One Health context. The observations showed that human-adapted strains might spill over to horses and, vice versa, that equine strains might colonize humans. This is of particular relevance in horse clinics. Therefore, it can be suggested to perform pre-surgical screening for *S. aureus* in both humans with horse contact as well as horses. In this context it would be an interesting topic to investigate the effect of the equine leukocidin on human cells as well as the pathogenicity of equine-adapted strains on humans and their possible persistence in human hosts. Further studies should investigate not only whether colonizations of horses by human strains, or of humans by equine strains, were permanent or transient, but also whether these strains are able to adapt to and cause invasive diseases in the new hosts.

## Figures and Tables

**Table 1:** MIC distributions of the 91 *S. aureus* isolates.

Antimicrobial Agents	MIC in (µg/mL)																		S	I	R
	0.008	0.015	0.03	0.06	0.12	0.25	0.5	1	2	4	8	16	32	64	128	256	512	1024	(µg/mL)		
**Oxacillin (total)**		-	-	-	2	14	46	17	2	3	-	7									
Equine		-	-	-	-	1	10 + 3	4 + 1	1	1 + 2	-	4							≤2	-	≥4
Human		-	-	-	1 + 1	12 + 1	29 + 4	9 + 3	1	-	-	3							≤2	-	≥4
**Penicillin (total)**		3	14	2	-	-	-	1	10	17	8	24	8	4							
Equine		1	2	-	-	-	-	-	2	3 + 1	3 + 1	4 + 1	5	3 + 1					≤0.5	1	≥2
Human		1 + 1	7 + 5	2	-	-	-	1	8	10 + 3	4	19	3						≤0.12	-	≥0.25
**Ampicillin (total)**			-	2	16	1	1	-	16	12	22	8	11	2	-						
Equine			-	2	1	-	-	-	3	2	5 + 2	2 + 1	7	1 + 1	-				≤0.25	0.5	≥1
Human			-	-	9 + 6	1	1	-	13	7 + 3	15	5	4	-	-						
**Amoxicillin/Clavulanic Acid (total)**			-	-	2	17	19	35	11	-	-	6	1	-	-						
Equine			-	-	1	2	3	10 + 2	3 + 2	-	-	3	1								
Human			-	-	1	10 + 5	16	22 + 1	4 + 2	-	-	3	-	-	-						
**Imipenem (total)**		31	53	-	-	-	-	3	-	1	3	-	-	-							
Equine		7 + 2	9 + 5	-	-	-	-	-	-	1	3	-	-	-							
Human		17 + 5	35 + 4	-	-	-	-	3	-	-	-	-	-	-							
**Ceftiofur (total)**			-	-	-	-	-	58	25	1	-	-	-	3	4						
Equine			-	-	-	-	-	14 + 4	2 + 3	-	-	-	-	-	4						
Human			-	-	-	-	-	33 + 7	18 + 2	1	-	-	-	3	-						
**Cefquinome (total)**		-	-	-	1	-	34	47	2	-	1	5	1	-							
Equine		-	-	-	1	-	8 + 3	7 + 3	1	-	-	3	1	-							
Human		-	-	-	-	-	19 + 4	32 + 5	1	-	1	2	-	-							
**Cefalothin (total)**				1	2	18	56	7	-	-	-	3	3	1	-	-					
Equine				1	1	1	13 + 3	2 + 2	-	-	-	-	3	1	-	-					
Human				-	1	12 + 5	36 + 4	3	-	-	-	3	-	-	-	-					
**Cefotaxime (total)**		-	-	-	-	-	-	-	33	50	1	-	-	7							
Equine		-	-	-	-	-	-	-	4 + 2	11 + 5	1			4							
Human		-	-	-	-	-	-	-	23 + 4	29 + 5		-	-	3							
**Cefoperazone (total)**				-	-	-	-	-	21	52	11	-	-	7							
Equine				-	-	-	-	-	1 + 3	12 + 1	3 + 3	-	-	4							
Human				-	-	-	-	-	12 + 5	35 + 4	5	-	-	3							
**Erythromycin (total)**		-	-	-	-	9	70	3	-	-	-	-	-	9							
Equine		-	-	-	-	5 + 3	11 + 4	-	-	-	-	-	-	4					≤0.5	1–4	≥8
Human		-	-	-	-	1	47 + 8	3	-	-	-	-	-	5					≤0.5	1–4	≥8
**Tylosin tartrate (total)**				-	-	-	-	5	67	19	-	-	-	-	-	-					
Equine				-	-	-	-	3 + 2	15 + 5	2	-	-	-	-	-	-					
Human				-	-	-	-	-	38 + 9	17	-	-	-	-	-	-					
**Tulathromycin (total)**				-	-	-	-	-	1	11	56	17	4	2							
Equine				-	-	-	-	-	1	2 + 4	12 + 3	1	4	-							
Human				-	-	-	-	-	-	1 + 4	36 + 5	16	-	2							
**Tilmicosin (total)**				-	-	1	5	48	37	-	-	-	-	-	-	-					
Equine				-	-	1	3 + 2	7 + 5	9	-	-	-	-	-	-						
Human				-	-	-	-	28 + 8	27 + 1	-	-	-	-	-	-						
**Clindamycin (total)**			-	2	22	66	1	-	-	-	-	-	-	-	-						
Equine			-	1 + 1	6 + 2	12 + 4	1	-	-	-	-	-	-	-	-						
Human			-	-	14	41 + 9	-	-	-	-	-	-	-	-	-				≤0.5	1–2	≥4
**Pirlimycin (total)**			-	-	-	2	22	66	1	-	-	-	-	-	-						
Equine			-	-	-	2	9 + 3	9 + 4	-	-	-	-	-	-	-						
Human			-	-	-	-	10	45 + 8	1	-	-	-	-	-	-						
**Tiamulin (total)**			-	-	1	1	13	74	2	-	-	-	-	-	-						
Equine			-	-	1	1	2 + 3	17 + 3	-	-	-	-	-	-	-						
Human			-	-	-	-	8	45 + 9	2	-	-	-	-	-	-						
**Ciprofloxacin (total)**	-	-	1	3	37	24	8	18	-	-	-	-	-								
Equine	-	-	1	1	9 + 4	7 + 2	-	3	-	-	-	-	-								
Human	-	-	-	1 + 1	18 + 6	14 + 1	7 + 1	15	-	-	-	-	-						≤1	2	≥4
**Enrofloxacin (total)**	-	-	3	40	24	7	17	-	-	-	-	-	-								
Equine	-	-	1 + 1	9 + 6	7	-	3	-	-	-	-	-	-						≤0.12	0.25	≥0.5
Human	-	-	1	19 + 6	14 + 3	7	14	-	-	-	-	-	-								
**Marbofloxacin (total)**	-	-	-	-	15	48	18	10	-	-	-	-	-								
Equine	-	-	-	-	5 + 1	8 + 6	5	2	-	-	-	-	-								
Human	-	-	-	-	8 + 1	27 + 7	12 + 1	8	-	-	-	-	-								
**Gentamicin (total)**					1	27	25	5	1	-	1	2	13	14	2	-	-				
Equine					-	4 + 2	4 + 1	1	1	-	-	-	2 + 3	6	2	-	-		≤4	8	≥16
Human					1	18 + 3	16 + 4	4	-	-	1	2	8	7 + 1	-	-	-		≤4	8	≥16
**Streptomycin (total)**						-	-	-	-	12	56	11	1	-	1	10	-	-			
Equine						-	-	-	-	2 + 3	10 + 3	4	-	-	1	4	-	-			
Human						-	-	-	-	6 + 1	36 + 7	7	1	-	-	6	-	-			
**Neomycin (total)**					-	4	40	12	5	7	8	6	7	2	-						
Equine					-	1	8 + 5	1	1	2	1 + 1	2	4	1	-						
Human					-	1 + 2	23 + 4	9 + 2	4	5	5 + 1	4	3	1	-						
**Tetracycline (total)**					2	43	16	-	-	-	-	2	21	7	-	-	-				
Equine					1	6 + 5	3 + 1	-	-	-	-	2	8 + 1	-	-	-	-		≤4	8	≥16
Human					1	26 + 6	10 + 2	-	-	-	-	-	11 + 1	7	-	-	-		≤4	8	≥16
**Doxycycline (total)**				3	33	19	6	-	10	19	1	-	-	-	-	-					
Equine				1 + 1	7 + 3	2 + 2	-	-	6 + 1	4	-	-	-	-	-	-			≤0.12	0.25	≥0.5
Human				1	19 + 4	13 + 2	5 + 1	-	3	14 + 1	1	-	-	-	-	-			≤4	8	≥16
**Sulfamethoxazole/trimethoprim (total)**		-	3	48	9	7	1	3	1	6	12	1	-	-							
Equine		-	2	9 + 1	1 + 3	4	-	2 + 1	1	-	3	-	-	-					≤2/38	-	≥4/76
Human		-	1	31 + 7	4 + 1	3	1	-	-	6	9	1	-	-					≤2/38	-	≥4/76
**Florfenicol (total)**					-	-	-	-	2	69	19	-	-	1	-	-	-				
Equine					-	-	-	-	1 + 1	17 + 6	2	-	-	-	-	-	-				
Human					-	-	-	-	-	39 + 7	15 + 2	-	-	1	-	-	-				
**Linezolid (total)**			-	-	-	-	-	8	62	21	-	-	-	-	-						
Equine			-	-	-	-	-	2 + 3	17 + 4	1	-	-	-	-	-				≤4	-	≥8
Human			-	-	-	-	-	3	36 + 5	16 + 4	-	-	-	-	-				≤4	-	≥8
**Vancomycin (total)**		-	-	-	-	-	6	83	2	-	-	-	-	-							
Equine		-	-	-	-	-	3	20 + 4	-	-	-	-	-	-							
Human		-	-	-	-	-	1 + 2	52 + 7	2	-	-	-	-	-					≤2	4–8	≥16
**Quinupristin/Dalfopristin (total)**		-	-	-	-	18	70	3	-	-	-	-	-	-							
Equine		-	-	-	-	4 + 4	16 + 3	-	-	-	-	-	-	-							
Human		-	-	-	-	6 + 4	46 + 5	3	-	-	-	-	-	-					≤1	2	≥4

S = susceptible; I = intermediate; R = resistant. Dark gray shading indicates concentrations not included in the test panel. Isolates with growth throughout the panel have MIC values equal to or larger than the highest concentration tested and are, therefore, displayed in the next higher concentration with gray shading. Amoxicillin and trimethoprim MIC values were used for the combinations amoxicillin/clavulanic acid (2:1) and sulfamethoxazole/trimethoprim (19:1), respectively. Light gray shading indicates the adoption of human clinical breakpoints for veterinary use. For the distributions of the equine and human isolates, CC1 isolates are displayed in purple, and CC1660 isolates are displayed in blue. In cases with clinical breakpoints, the start of an intermediate category is indicated by yellow vertical bars and the start of a resistant category is indicated by red vertical bars.

**Table 2:** Phenotypic and genotypic resistance patterns of the 91 *S. aureus* isolates.

Isolates Phenotype Total	Isolates Genotype Total	CC1 Horses	CC1 Humans	CC1660 Horses	CC1660 Humans	PEN	OXA	TET	GEN	ERY	SXT	FQ	NEO	STR	FFN
18	16	-	8	3	5										
	2		2	-	-						*folP* (F17L)				
31	32	9	19	-	3	*blaZ*									
1		1	-	-	-	*blaZ*									
1	1	-	1	-	-	*blaZ*									*fexA*
2	2	-	2	-	-	*blaZ*					*dfrG + folP* (F17L and A184V)				
2	2	-	2	-	-	*blaZ*				*erm*(C)				*str*	
3	1	-	1	-	-	*blaZ*			*aacA-aphD *		*dfrS1*	*grlA* (S80Y)			
	4	-	1	1	-	*blaZ*			*aacA-aphD *		*dfrS1*				
1		-	-	1	-	*blaZ*			*aacA-aphD *		*dfrS1*				
1		-	-	1	-	*blaZ*			*aacA-aphD*		*dfrS1*				
1	1	-	1	-	-	*blaZ*			*aacA-aphD *		*dfrS1/dfrG + folP * (F17L and A184V)				
1	1	-	1	-	-	*blaZ*		*tet*(K)					*aphA3*	*aad*(E)	
1	1	-	-	-	1			*tet*(L)	*aacA-aphD *		*dfrS1*		*aadD *		
4	3	3	-	-	-	*blaZ*		*tet*(L)	*aacA-aphD *		*dfrK*		*aadD *		
	1	-	1	-	-	*blaZ*		*tet*(L)	*aacA-aphD *		*dfrG*		*aadD *		
1	1	-	-	1	-	*blaZ*		*tet*(L)	*aacA-aphD *		*dfrK*		*aadD *	*str*	
3	3	-	3	-	-	*blaZ*	*mecA*	*tet*(K)		*erm*(C)			*aphA3*	*aad*(E)	
13	2	-	2	-	-	*blaZ*		*tet*(L)	*aacA-aphD *		*dfrG + folP * (F17L and A184V)		*aadD *		
	1	-	1	-	-	*blaZ*		*tet*(L)	*aacA-aphD *		*dfrS1/dfrG + folP * (F17L and A184V)	*grlA* (S80Y)	*aadD *		
	13	-	10	-	-	*blaZ*		*tet*(L)	*aacA-aphD *		*dfrG + folP * (F17L and A184V)	*grlA* (S80Y)	*aadD *		
3		3	-	-	-	*blaZ*		*tet*(L)	*aacA-aphD *		*dfrG + folP * (F17L and A184V)	*grlA* (S80Y)	*aadD*		
4	4	4	-	-	-	*blaZ*	*mecA*	*tet*(K)	*aacA-aphD *	*erm*(C)	*dfrS1*		*aphA3*	*aad*(E)	

Abbreviations: PEN = penicillins; OXA = oxacillin; TET = tetracyclines; GEN = gentamicin; ERY = erythromycin; SXT = sulfamethoxazole/trimethoprim; FQ = fluoroquinolones; NEO = neomycin; STR = streptomycin; FFN = florfenicol; red shading = phenotypic resistance; genes/mutations = presence of genetic resistance determinants. Fluoroquinolone resistance was determined based on clinical breakpoints for ciprofloxacin (humans) and enrofloxacin (equine/veterinarian), respectively. For some antimicrobial agents without clinical breakpoints, “resistance” was classified based on the MICs as follows: neomycin ≥ 4 µg/mL, streptomycin ≥ 128 µg/mL, florfenicol ≥ 64 µg/mL.

**Table 3:** Distribution of virulence genes among the 75 CC1 and 16 CC1660 isolates.

Virulence Genes	Localization	In CC1 Isolates (*n* = 75)	In CC1660 Isolates (*n* = 16)	In Human Isolates (*n* = 67)	In Equine Isolates (*n* = 24)
*tst-1 + sec + sel*	SaPI	0	3 (18.75%)	3 (4.48%)	0
*seb*	SaPI	4 (5.33%)	0	4 (5.97%)	0
*seb + sek + seq*	SaPI	4 (5.33%)	0	4 (5.97%)	0
*sek + seq*	SaPI	1 (1.33%)	0	1 (1.49%)	0
*scn2 + vwb3*	SaPI	49 (65.33%)	16 (100%)	42 (62.69%)	23 (95.83%)
*sea + sak + scn*	prophage	7 (9.33%)	0	7 (10.45%)	0
*sak + scn*	prophage	8 (10.67%)	0	4 (5.97%)	4 (16.67%)
*lukP + lukQ + scn-eq*	prophage	46 (61.33%)	14 (87.50%)	38 (56.72%)	22 (91.67%)
*sak_phi-42e*	prophage	46 (61.33%)	13 (81.25%)	41 (61.19%)	18 (75%)

## Data Availability

This Whole Genome Shotgun project has been deposited at DDBJ/ENA/GenBank under BioProject Accession number PRJNA1263585. The individual accession numbers (BioSample, Genome, SRA) are displayed in Table S1.

## Supplementary Materials

The following supporting information can be downloaded at https://www.mdpi.com/article/10.3390/antibiotics14111082/s1. Figure S1: Phenotypically different isolates within the samples; Figure S2: Geographic origin of the samples included; Figure S3: Distribution of the isolates from the different equine clinics (EC); Figure S4: Genetic relationship of the s*pa* types detected among the CC1 (a) and CC1660 (b) isolates; Figure S5: *arcC*-PCR; Figure S6: Testing of the anaerobic arginine dehydrolase activity; Table S1: Background information of the 91 *S. aureus* isolates; Table S2: Heatmap of the cgMLST; Table S3: Primers used for MLST of *Staphylococcus aureus* according to reference [5].

## Abbreviations

The following abbreviations are used in this manuscript:

BORSA	borderline oxacillin-resistant *Staphylococcus aureus*
bp	base pair
CC	clonal complex
EC	equine clinic
*dru*	direct repeat unit
MIC	minimal inhibitory concentration
MLST	multilocus sequence typing
MRSA	methicillin-resistant *Staphylococcus aureus*
MSSA	methicillin-susceptible *Staphylococcus aureus*
QRDR	quinolone resistance-determining region
*S.*	*Staphylococcus*
SaPI	staphylococcal pathogenicity island
SCC	Staphylococcal Cassette Chromosome
SNP	single nucleotide polymorphism
*spa*	staphylococcal protein A
ST	sequence type
WGS	whole-genome sequencing

## References

[1] Rasquel-Oliveira F.S., Ribeiro J.M., Martelossi-Cebinelli G., Costa F.B., Nakazato G., Casagrande R., Verri W.A. (2025). *Staphylococcus aureus* in Inflammation and Pain: Update on Pathologic Mechanisms. Pathogens.

[2] Holmes M.A., Zadoks R.N. (2011). Methicillin resistant *S. aureus* in human and bovine mastitis. J. Mammary Gland Biol. Neoplasia.

[3] Cuny C., Friedrich A., Kozytska S., Layer F., Nübel U., Ohlsen K., Strommenger B., Walther B., Wieler L., Witte W. (2010). Emergence of methicillin-resistant *Staphylococcus aureus* (MRSA) in different animal species. Int. J. Med. Microbiol..

[4] Marshall K., Marsella R. (2023). Evolution of the Prevalence of Antibiotic Resistance to *Staphylococcus* spp. Isolated from Horses in Florida over a 10-Year Period. Vet. Sci..

[5] Enright M.C., Day N.P., Davies C.E., Peacock S.J., Spratt B.G. (2000). Multilocus sequence typing for characterization of methicillin-resistant and methicillin-susceptible clones of *Staphylococcus aureus*. J. Clin. Microbiol..

[6] Feil E.J., Cooper J.E., Grundmann H., Robinson D.A., Enright M.C., Berendt T., Peacock S.J., Smith J.M., Murphy M., Spratt B.G. (2003). How clonal is *Staphylococcus aureus*?. J. Bacteriol..

[7] Lindsay J.A., Moore C.E., Day N.P., Peacock S.J., Witney A.A., Stabler R.A., Husain S.E., Butcher P.D., Hinds J. (2006). Microarrays reveal that each of the ten dominant lineages of *Staphylococcus aureus* has a unique combination of surface-associated and regulatory genes. J. Bacteriol..

[8] Jolley K., Bray J., Maiden M. (2018). Open-access bacterial population genomics: BIGSdb software, the PubMLST.org website and their applications. Wellcome Open Res..

[9] Monecke S., Luedicke C., Slickers P., Ehricht R. (2009). Molecular epidemiology of *Staphylococcus aureus* in asymptomatic carriers. Eur. J. Clin. Microbiol. Infect. Dis..

[10] Holtfreter S., Grumann D., Balau V., Barwich A., Kolata J., Goehler A., Weiss S., Holtfreter B., Bauerfeind S.S., Döring P. (2016). Molecular Epidemiology of *Staphylococcus aureus* in the General Population in Northeast Germany: Results of the Study of Health in Pomerania (SHIP-TREND-0). J. Clin. Microbiol..

[11] Rao Q., Shang W., Hu X., Rao X. (2015). *Staphylococcus aureus* ST121: A globally disseminated hypervirulent clone. J. Med. Microbiol..

[12] Monecke S., Feßler A.T., Burgold-Voigt S., Krüger H., Mühldorfer K., Wibbelt G., Liebler-Tenorio E.M., Reinicke M., Braun S.D., Hanke D. (2021). *Staphylococcus aureus* isolates from Eurasian Beavers (*Castor fiber*) carry a novel phage-borne bicomponent leukocidin related to the Panton-Valentine leukocidin. Sci. Rep..

[13] Moon D.C., Kim B.Y., Tamang M.D., Nam H.M., Jang G.C., Jung S.C., Lee H.S., Park Y.H., Lim S.K. (2016). Genome Sequence of a Unique t2247-ST692-III Livestock-Associated Methicillin-Resistant *Staphylococcus aureus* Strain from Chicken Carcass. Genome Announc..

[14] Monecke S., Gavier-Widén D., Hotzel H., Peters M., Guenther S., Lazaris A., Loncaric I., Müller E., Reissig A., Ruppelt-Lorz A. (2016). Diversity of *Staphylococcus aureus* Isolates in European Wildlife. PLoS ONE.

[15] García-Álvarez L., Holden M.T., Lindsay H., Webb C.R., Brown D.F., Curran M.D., Walpole E., Brooks K., Pickard D.J., Teale C. (2011). Meticillin-resistant *Staphylococcus aureus* with a novel *mecA* homologue in human and bovine populations in the UK and Denmark: A descriptive study. Lancet Infect. Dis..

[16] Vancraeynest D., Haesebrouck F., Deplano A., Denis O., Godard C., Wildemauwe C., Hermans K. (2006). International dissemination of a high virulence rabbit *Staphylococcus aureus* clone. J. Vet. Med. B Infect. Dis. Vet. Public Health.

[17] Burgold-Voigt S., Monecke S., Busch A., Bocklisch H., Braun S.D., Diezel C., Hotzel H., Liebler-Tenorio E.M., Müller E., Reinicke M. (2023). Characterisation of a *Staphylococcus aureus* Isolate Carrying Phage-Borne Enterotoxin E from a European Badger (*Meles meles*). Pathogens.

[18] Bar-Gal G.K., Blum S.E., Hadas L., Ehricht R., Monecke S., Leitner G. (2015). Host-specificity of *Staphylococcus aureus* causing intramammary infections in dairy animals assessed by genotyping and virulence genes. Vet. Microbiol..

[19] Smyth D.S., Feil E.J., Meaney W.J., Hartigan P.J., Tollersrud T., Fitzgerald J.R., Enright M.C., Smyth C.J. (2009). Molecular genetic typing reveals further insights into the diversity of animal-associated *Staphylococcus aureus*. J. Med. Microbiol..

[20] Schlotter K., Ehricht R., Hotzel H., Monecke S., Pfeffer M., Donat K. (2012). Leukocidin genes *lukF-P83* and *lukM* are associated with *Staphylococcus aureus* clonal complexes 151, 479 and 133 isolated from bovine udder infections in Thuringia, Germany. Vet. Res..

[21] Richardson E.J., Bacigalupe R., Harrison E.M., Weinert L.A., Lycett S., Vrieling M., Robb K., Hoskisson P.A., Holden M.T.G., Feil E.J. (2018). Gene exchange drives the ecological success of a multi-host bacterial pathogen. Nat. Ecol. Evol..

[22] Voss A., Loeffen F., Bakker J., Klaassen C., Wulf M. (2005). Methicillin-resistant *Staphylococcus aureus* in pig farming. Emerg. Infect. Dis..

[23] Monecke S., Kuhnert P., Hotzel H., Slickers P., Ehricht R. (2007). Microarray based study on virulence-associated genes and resistance determinants of *Staphylococcus aureus* isolates from cattle. Vet. Microbiol..

[24] Witte W., Strommenger B., Stanek C., Cuny C. (2007). Methicillin-resistant *Staphylococcus aureus* ST398 in humans and animals, Central Europe. Emerg. Infect. Dis..

[25] Nemati M., Hermans K., Lipinska U., Denis O., Deplano A., Struelens M., Devriese L.A., Pasmans F., Haesebrouck F. (2008). Antimicrobial resistance of old and recent *Staphylococcus aureus* isolates from poultry: First detection of livestock-associated methicillin-resistant strain ST398. Antimicrob. Agents Chemother..

[26] Cuny C., Abdelbary M.M.H., Köck R., Layer F., Scheidemann W., Werner G., Witte W. (2016). Methicillin-resistant *Staphylococcus aureus* from infections in horses in Germany are frequent colonizers of veterinarians but rare among MRSA from infections in humans. One Health.

[27] Murphy R.J.T., Ramsay J.P., Lee Y.T., Pang S., O’Dea M.A., Pearson J.C., Axon J.E., Raby E., Abdulgader S.M., Whitelaw A. (2019). Multiple introductions of methicillin-resistant *Staphylococcus aureus* ST612 into Western Australia associated both with human and equine reservoirs. Int. J. Antimicrob. Agents.

[28] Cuny C., Witte W. (2017). MRSA in equine hospitals and its significance for infections in humans. Vet. Microbiol..

[29] Albrecht N., Jatzwauk L., Slickers P., Ehricht R., Monecke S. (2011). Clonal replacement of epidemic methicillin-resistant *Staphylococcus aureus* strains in a German university hospital over a period of eleven years. PLoS ONE.

[30] Merz A., Stephan R., Johler S. (2016). *Staphylococcus aureus* Isolates from Goat and Sheep Milk Seem to Be Closely Related and Differ from Isolates Detected from Bovine Milk. Front. Microbiol..

[31] Azara E., Piras M.G., Parisi A., Tola S. (2017). Antimicrobial susceptibility and genotyping of *Staphylococcus aureus* isolates collected between 1986 and 2015 from ovine mastitis. Vet. Microbiol..

[32] Carfora V., Giacinti G., Sagrafoli D., Marri N., Giangolini G., Alba P., Feltrin F., Sorbara L., Amoruso R., Caprioli A. (2016). Methicillin-resistant and methicillin-susceptible *Staphylococcus aureus* in dairy sheep and in-contact humans: An intra-farm study. J. Dairy Sci..

[33] Monecke S., Burgold-Voigt S., Feßler A.T., Krapf M., Loncaric I., Liebler-Tenorio E.M., Braun S.D., Diezel C., Müller E., Reinicke M. (2025). Characterisation of *Staphylococcus aureus* Strains and Their Prophages That Carry Horse-Specific Leukocidin Genes *lukP/Q*. Toxins.

[34] Sieber S., Gerber V., Jandova V., Rossano A., Evison J.M., Perreten V. (2011). Evolution of multidrug-resistant *Staphylococcus aureus* infections in horses and colonized personnel in an equine clinic between 2005 and 2010. Microb. Drug Resist..

[35] Islam M.Z., Espinosa-Gongora C., Damborg P., Sieber R.N., Munk R., Husted L., Moodley A., Skov R., Larsen J., Guardabassi L. (2017). Horses in Denmark Are a Reservoir of Diverse Clones of Methicillin-Resistant and -Susceptible *Staphylococcus aureus*. Front. Microbiol..

[36] Sung J.M., Lloyd D.H., Lindsay J.A. (2008). *Staphylococcus aureus* host specificity: Comparative genomics of human versus animal isolates by multi-strain microarray. Microbiology.

[37] Monecke S., Coombs G., Shore A.C., Coleman D.C., Akpaka P., Borg M., Chow H., Ip M., Jatzwauk L., Jonas D. (2011). A field guide to pandemic, epidemic and sporadic clones of methicillin-resistant *Staphylococcus aureus*. PLoS ONE.

[38] Lawal O.U., Ayobami O., Abouelfetouh A., Mourabit N., Kaba M., Egyir B., Abdulgader S.M., Shittu A.O. (2022). A 6-Year Update on the Diversity of Methicillin-Resistant *Staphylococcus aureus* Clones in Africa: A Systematic Review. Front. Microbiol..

[39] Williamson D.A., Monecke S., Heffernan H., Ritchie S.R., Roberts S.A., Upton A., Thomas M.G., Fraser J.D. (2014). High usage of topical fusidic acid and rapid clonal expansion of fusidic acid-resistant *Staphylococcus aureus*: A cautionary tale. Clin. Infect. Dis..

[40] Alba P., Feltrin F., Cordaro G., Porrero M.C., Kraushaar B., Argudín M.A., Nykäsenoja S., Monaco M., Stegger M., Aarestrup F.M. (2015). Livestock-Associated Methicillin Resistant and Methicillin Susceptible *Staphylococcus aureus* Sequence Type (CC)1 in European Farmed Animals: High Genetic Relatedness of Isolates from Italian Cattle Herds and Humans. PLoS ONE.

[41] Leopold S.R., Goering R.V., Witten A., Harmsen D., Mellmann A. (2014). Bacterial whole-genome sequencing revisited: Portable, scalable, and standardized analysis for typing and detection of virulence and antibiotic resistance genes. J. Clin. Microbiol..

[42] Cunningham S.A., Chia N., Jeraldo P.R., Quest D.J., Johnson J.A., Boxrud D.J., Taylor A.J., Chen J., Jenkins G.D., Drucker T.M. (2017). Comparison of Whole-Genome Sequencing Methods for Analysis of Three Methicillin-Resistant *Staphylococcus aureus* Outbreaks. J. Clin. Microbiol..

[43] Graveland H., Wagenaar J.A., Bergs K., Heesterbeek H., Heederik D. (2011). Persistence of livestock associated MRSA CC398 in humans is dependent on intensity of animal contact. PLoS ONE.

[44] Ramón-Maiques S., Marina A., Guinot A., Gil-Ortiz F., Uriarte M., Fita I., Rubio V. (2010). Substrate binding and catalysis in carbamate kinase ascertained by crystallographic and site-directed mutagenesis studies: Movements and significance of a unique globular subdomain of this key enzyme for fermentative ATP production in bacteria. J. Mol. Biol..

[45] Zhang L., Thomas J.C., Didelot X., Robinson D.A. (2012). Molecular signatures identify a candidate target of balancing selection in an *arcD*-like gene of *Staphylococcus epidermidis*. J. Mol. Evol..

[46] Lodder G., Werckenthin C., Schwarz S., Dyke K. (1997). Molecular analysis of naturally occuring *erm*C-encoding plasmids in staphylococci isolated from animals with and without previous contact with macrolide/lincosamide antibiotics. FEMS Immunol. Med. Microbiol..

[47] EMA (European Medicines Agency) (2019). Categorisation of Antibiotics in the European Union.

[48] CLSI (2024). Performance Standards for Antimicrobial Disk and Dilution Susceptibility Tests for Bacteria Isolated from Animals.

[49] CLSI (2025). Performance Standards for Antimicrobial Susceptibility Testing.

[50] Guignard B., Entenza J.M., Moreillon P. (2005). Beta-lactams against methicillin-resistant *Staphylococcus aureus*. Curr. Opin. Pharmacol..

[51] Scholtzek A.D., Hanke D., Walther B., Eichhorn I., Stöckle S.D., Klein K.S., Gehlen H., Lübke-Becker A., Schwarz S., Feßler A.T. (2019). Molecular Characterization of Equine *Staphylococcus aureus* Isolates Exhibiting Reduced Oxacillin Susceptibility. Toxins.

[52] Feßler A., Scott C., Kadlec K., Ehricht R., Monecke S., Schwarz S. (2010). Characterization of methicillin-resistant *Staphylococcus aureus* ST398 from cases of bovine mastitis. J. Antimicrob. Chemother..

[53] Brennan G.I., Abbott Y., Burns A., Leonard F., McManus B.A., O’Connell B., Coleman D.C., Shore A.C. (2016). The Emergence and Spread of Multiple Livestock-Associated Clonal Complex 398 Methicillin-Resistant and Methicillin-Susceptible *Staphylococcus aureus* Strains among Animals and Humans in the Republic of Ireland, 2010–2014. PLoS ONE.

[54] Scholtzek A.D., Hanke D., Eichhorn I., Walther B., Lübke-Becker A., van Duijkeren E., Köck R., Schwarz S., Feßler A.T. (2020). Heterogeneity of antimicrobial susceptibility testing results for sulfamethoxazole/trimethoprim obtained from clinical equine *Staphylococcus aureus* isolates using different methods. Vet. Microbiol..

[55] Kehrenberg C., Schwarz S. (2004). *fexA*, a novel *Staphylococcus lentus* gene encoding resistance to florfenicol and chloramphenicol. Antimicrob. Agents Chemother..

[56] Goering R.V., Morrison D., Al-Doori Z., Edwards G.F., Gemmell C.G. (2008). Usefulness of *mec*-associated direct repeat unit (*dru*) typing in the epidemiological analysis of highly clonal methicillin-resistant *Staphylococcus aureus* in Scotland. Clin. Microbiol. Infect..

[57] Monecke S., König E., Earls M.R., Leitner E., Müller E., Wagner G.E., Poitz D.M., Jatzwauk L., Vremerǎ T., Dorneanu O.S. (2020). An epidemic CC1-MRSA-IV clone yields false-negative test results in molecular MRSA identification assays: A note of caution, Austria, Germany, Ireland, 2020. Euro Surveill..

[58] Cuny C., Strommenger B., Witte W., Stanek C. (2008). Clusters of infections in horses with MRSA ST1, ST254, and ST398 in a veterinary hospital. Microb. Drug Resist..

[59] Wick R.R., Judd L.M., Gorrie C.L., Holt K.E. (2017). Unicycler: Resolving bacterial genome assemblies from short and long sequencing reads. PLoS Comput. Biol..

[60] Prjibelski A., Antipov D., Meleshko D., Lapidus A., Korobeynikov A. (2020). Using SPAdes De Novo Assembler. Curr. Protoc. Bioinform..

[61] Seemann T. mlst. *Github*. https://github.com/tseemann/mlst.

[62] Bartels M.D., Petersen A., Worning P., Nielsen J.B., Larner-Svensson H., Johansen H.K., Andersen L.P., Jarløv J.O., Boye K., Larsen A.R. (2014). Comparing whole-genome sequencing with Sanger sequencing for *spa* typing of methicillin-resistant *Staphylococcus aureus*. J. Clin. Microbiol..

[63] Silva M., Machado M.P., Silva D.N., Rossi M., Moran-Gilad J., Santos S., Ramirez M., Carriço J.A. (2018). chewBBACA: A complete suite for gene-by-gene schema creation and strain identification. Microb. Genom..

[64] Zhou Z., Alikhan N.F., Sergeant M.J., Luhmann N., Vaz C., Francisco A.P., Carriço J.A., Achtman M. (2018). GrapeTree: Visualization of core genomic relationships among 100,000 bacterial pathogens. Genome Res..

[65] CLSI (2024). Performance Standards for Antimicrobial Disk and Dilution Susceptibility Tests for Bacteria Isolated from Animals.

[66] BVL (2020). BVL-Report 14.6 Bericht zur Resistenzmonitoringstudie 2018 [BVL-Report 14.6 Report on the Resistance Monitoring Study 2018].

[67] Feldgarden M., Brover V., Haft D.H., Prasad A.B., Slotta D.J., Tolstoy I., Tyson G.H., Zhao S., Hsu C.H., McDermott P.F. (2019). Validating the AMRFinder Tool and Resistance Gene Database by Using Antimicrobial Resistance Genotype-Phenotype Correlations in a Collection of Isolates. Antimicrob. Agents Chemother..

[68] Bortolaia V., Kaas R.S., Ruppe E., Roberts M.C., Schwarz S., Cattoir V., Philippon A., Allesoe R.L., Rebelo A.R., Florensa A.F. (2020). ResFinder 4.0 for predictions of phenotypes from genotypes. J. Antimicrob. Chemother..

[69] Zankari E., Allesøe R., Joensen K.G., Cavaco L.M., Lund O., Aarestrup F.M. (2017). PointFinder: A novel web tool for WGS-based detection of antimicrobial resistance associated with chromosomal point mutations in bacterial pathogens. J. Antimicrob. Chemother..

[70] Altschul S.F., Gish W., Miller W., Myers E.W., Lipman D.J. (1990). Basic local alignment search tool. J. Mol. Biol..

[71] Consortium T.U. (2024). UniProt: The Universal Protein Knowledgebase in 2025. Nucleic Acids Res..

[72] Chen L., Yang J., Yu J., Yao Z., Sun L., Shen Y., Jin Q. (2005). VFDB: A reference database for bacterial virulence factors. Nucleic Acids Res..

[73] Monecke S., Gavier-Widen D., Mattsson R., Rangstrup-Christensen L., Lazaris A., Coleman D.C., Shore A.C., Ehricht R. (2013). Detection of *mecC*-positive *Staphylococcus aureus* (CC130-MRSA-XI) in diseased European hedgehogs (*Erinaceus europaeus*) in Sweden. PLoS ONE.

[74] Monecke S., Jatzwauk L., Müller E., Nitschke H., Pfohl K., Slickers P., Reissig A., Ruppelt-Lorz A., Ehricht R. (2016). Diversity of SCC*mec* Elements in *Staphylococcus aureus* as Observed in South-Eastern Germany. PLoS ONE.

[75] Coleman D., Knights J., Russell R., Shanley D., Birkbeck T.H., Dougan G., Charles I. (1991). Insertional inactivation of the *Staphylococcus aureus* beta-toxin by bacteriophage phi 13 occurs by site- and orientation-specific integration of the phi 13 genome. Mol. Microbiol..

[76] Coleman D.C., Sullivan D.J., Russell R.J., Arbuthnott J.P., Carey B.F., Pomeroy H.M. (1989). *Staphylococcus aureus* Bacteriophages Mediating the Simultaneous Lysogenic Conversion of *β*-Lysin, Staphylokinase and Enterotoxin A: Molecular Mechanism of Triple Conversion. J. Gen. Microbiol..

