# Supplementary material for: Characterization of Staphylococcus aureus CC1 and CC1660 of Human and Equine Origin
Source: Antibiotics (Basel). 2025 Oct 27;14(11):1082. doi: 10.3390/antibiotics14111082 (PMC12649262; doi:10.3390/antibiotics14111082)
Supplement: Supplementary file 1 [file antibiotics-14-01082-s001.zip › Table S3.pdf]

Table S3: Primers used for MLST of *Staphylococcus aureus* according to reference [5]

Gene	Function	Primer sequence (5' → 3')	Amplicon size (bp)
arcC	Carbamatekinase	Forward: TTGATTCACCAGCGCGTATTGTC<br>Reverse: AGGTATCTGCTTCAATCAGCG	456
aroE	Shikimatedehydrogenase	Forward: ATCGGAAATCCTATTTCACATTC<br>Reverse: GGTGTTGTATTAATAACGATATC	456
glpF	Glycerolkinase	Forward: CTAGGAACTGCAATCTTAATCC<br>Reverse: TGGTAAAATCGCATGTCCAATTC	465
gmk	Guanylatekinase	Forward: ATCGTTTTATCGGGACCATC<br>Reverse: TCATTAACTACAACGTAATCGTA	429
pta	Phosphateacetyltransferase	Forward: GTTAAAATCGTATTACCTGAAGG<br>Reverse: GACCCTTTTGTTGAAAAGCTTAA	474
tpi	Triosephosphateisomerase	Forward: TCGTTCATTCTGAACGTCGTGAA<br>Reverse: TTTGCACCTTCTAACAATTGTAC	402
yqiL	AcetylcoenzymeAacetyltransferase	Forward: CAGCATACAGGACACCTATTGGC<br>Reverse: CGTTGAGGAATCGATACTGGAAC	516

The *S. aureus* MLST scheme uses internal fragments of the seven house-keeping genes listed in the supplemental table S3.

According to the information given on https://pubmlst.org/organisms/staphylococcus-aureus/primers, PCR amplification is carried out on chromosomal DNA using an extension time of 30s, and an annealing temperature of 55°C, with Qiagen Taq polymerase. As the same primers are used for amplification and sequencing, it is important that only a single DNA fragment is amplified in the initial PCR. This may involve some optimization of the annealing temperature.
