# Supplementary material for: Characterization of Staphylococcus aureus CC1 and CC1660 of Human and Equine Origin
Source: Antibiotics (Basel). 2025 Oct 27;14(11):1082. doi: 10.3390/antibiotics14111082 (PMC12649262; doi:10.3390/antibiotics14111082)
Supplement: Supplementary file 1 [file antibiotics-14-01082-s001.zip › Sammelmappe1.pdf]

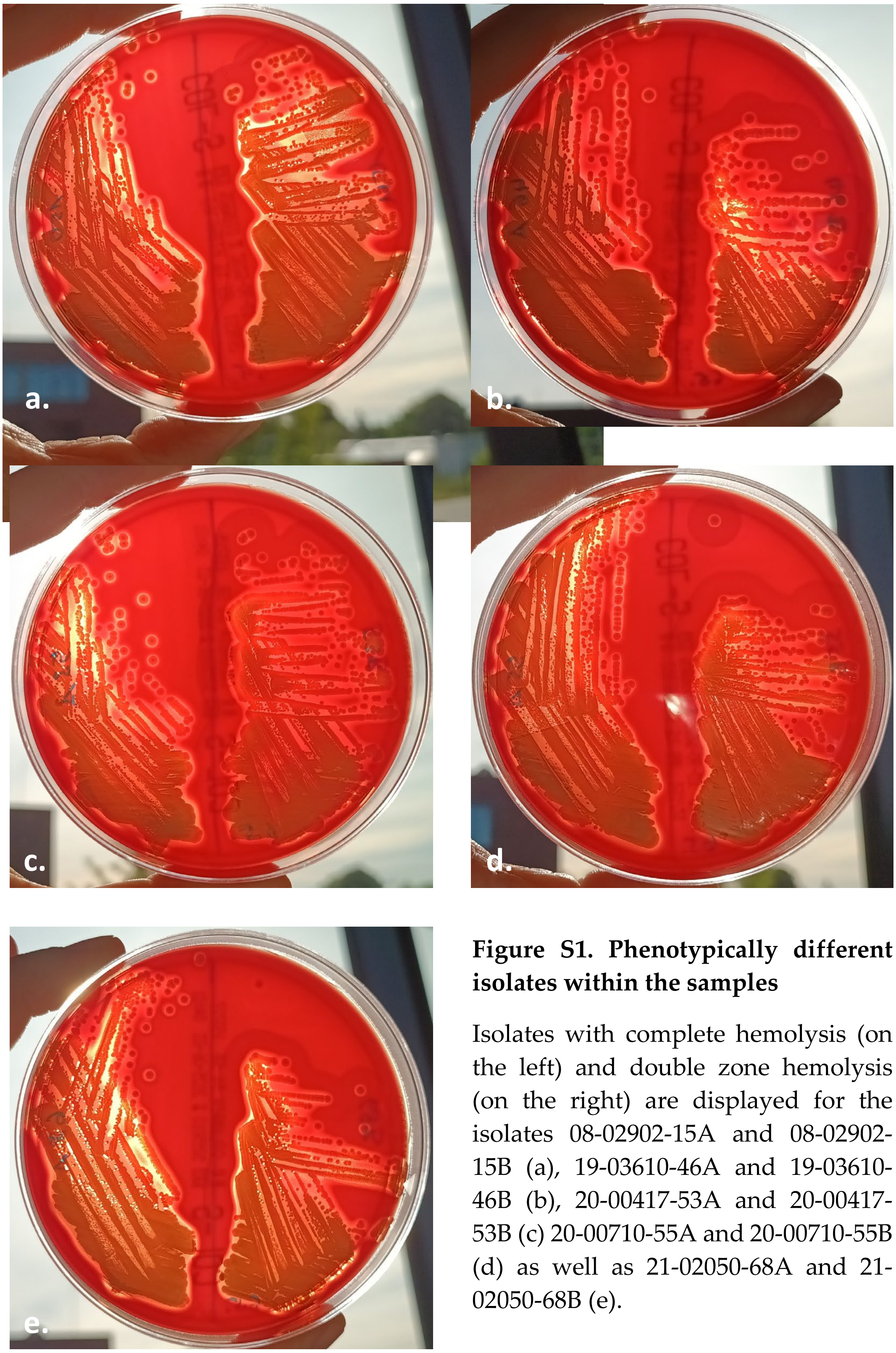

Figure S1. Phenotypically different isolates within the samples

Isolates with complete hemolysis (on the left) and double zone hemolysis (on the right) are displayed for the isolates 08-02902-15A and 08-02902-15B (a), 19-03610-46A and 19-03610-46B (b), 20-00417-53A and 20-00417-53B (c) 20-00710-55A and 20-00710-55B (d) as well as 21-02050-68A and 21-02050-68B (e).

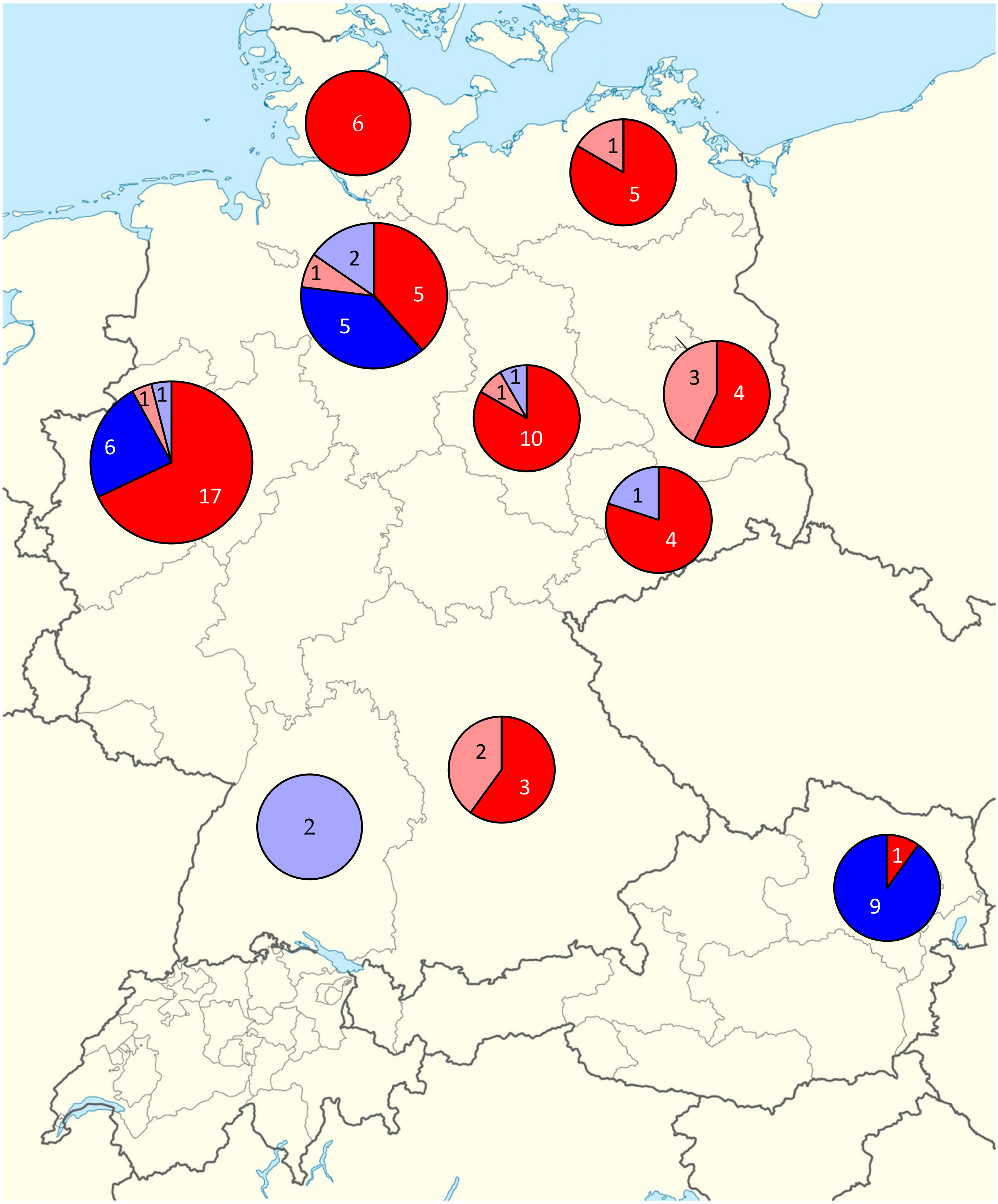

Figure S2. Geograpic origin of the samples included

human CC1 equine CC1
human CC1660 equine CC1660

the numbers of the respective isolates are given as numbers in the respective parts of the diagrams
the file was generated using the map from https://de.m.wikipedia.org/wiki/Datei:D-A-CH_location_map.svg#file

a)

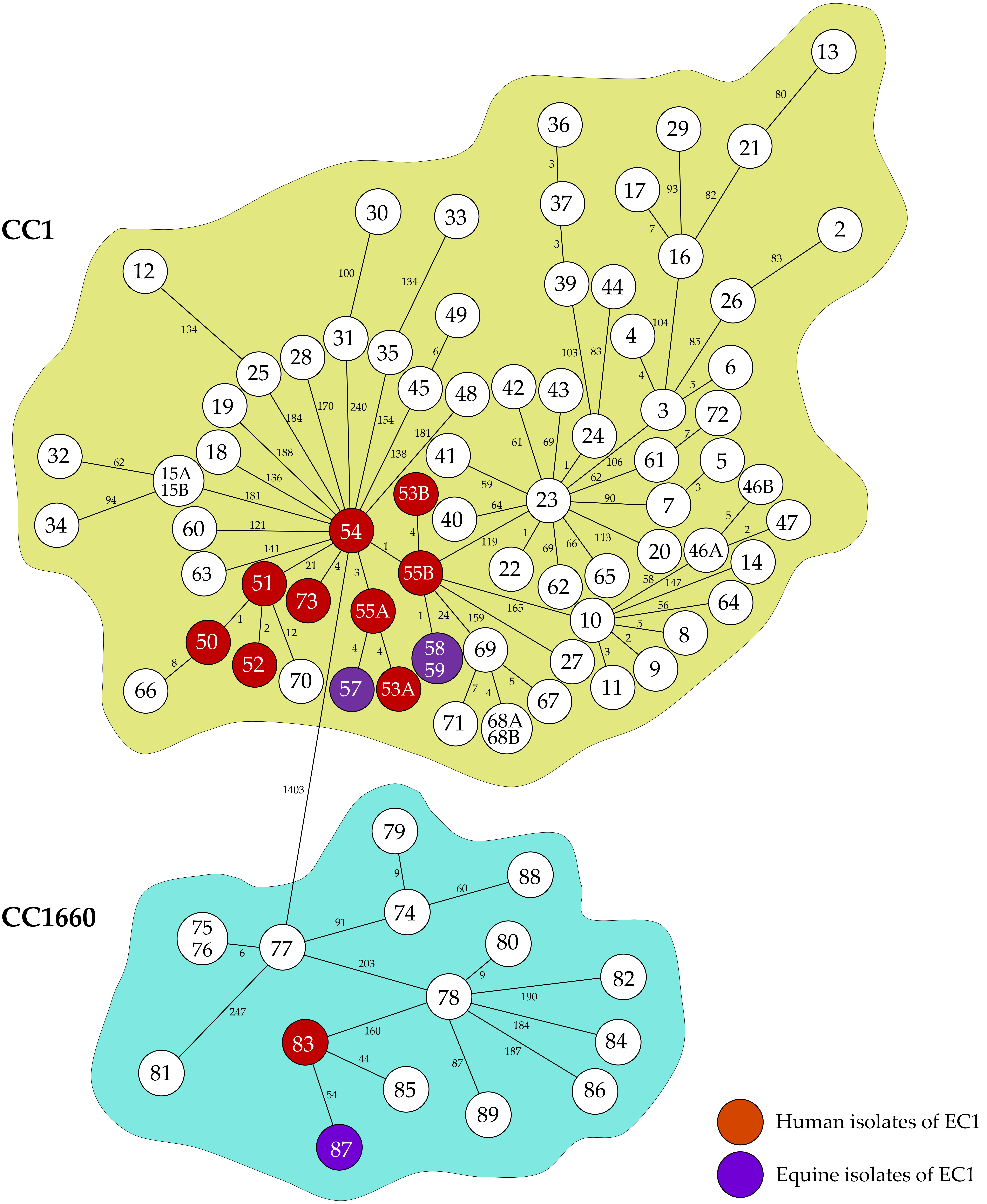

b)

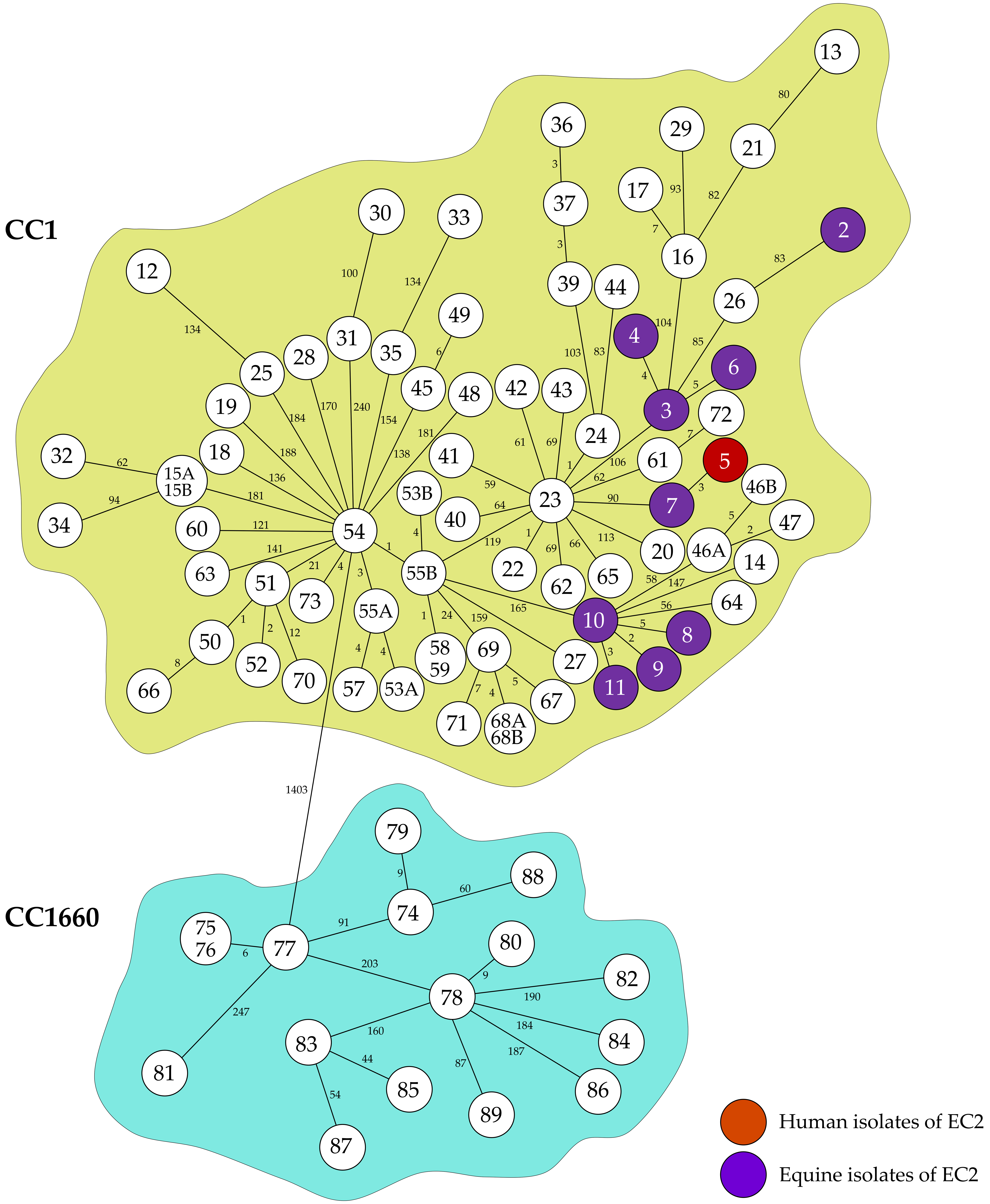

c)

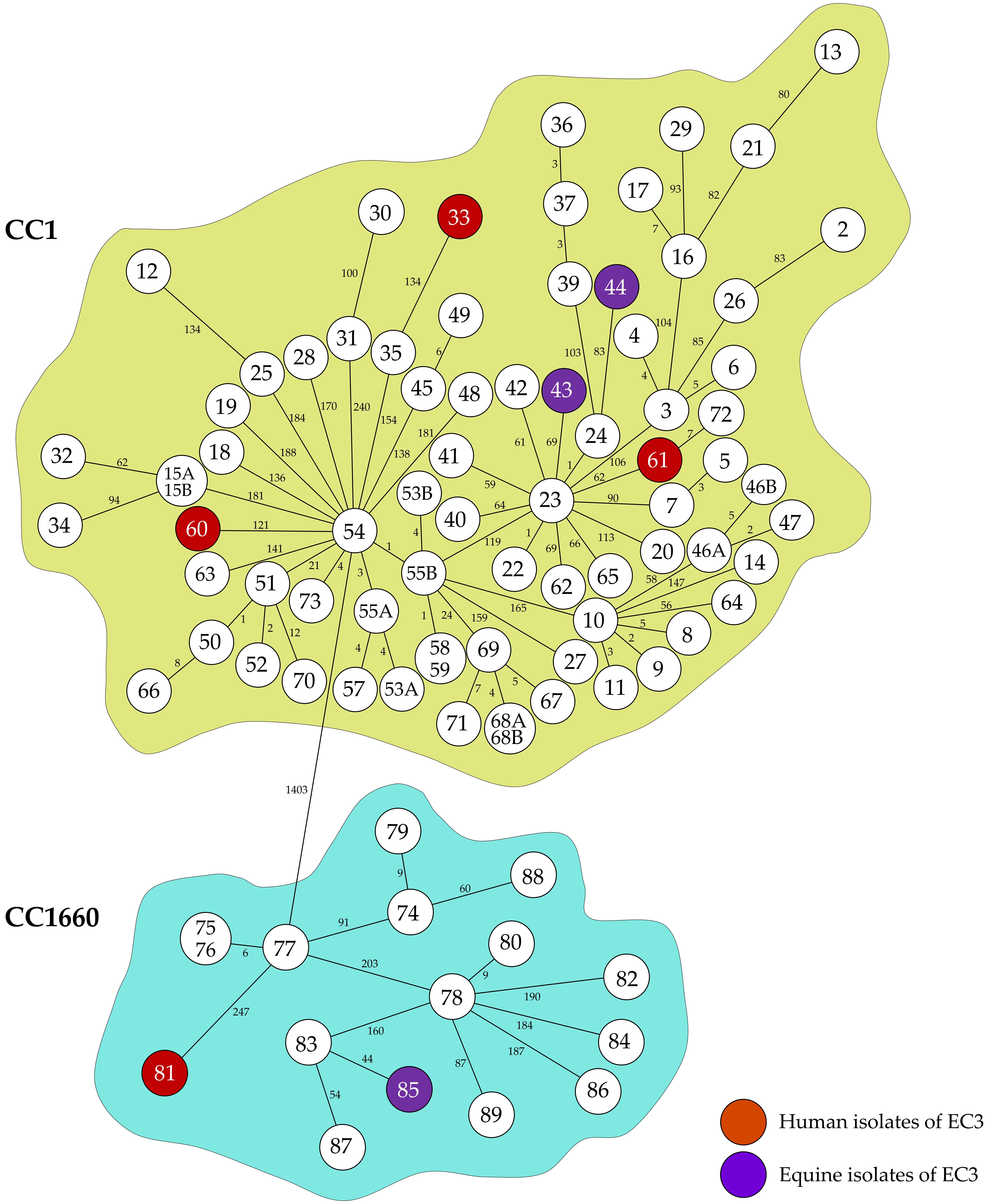

d)

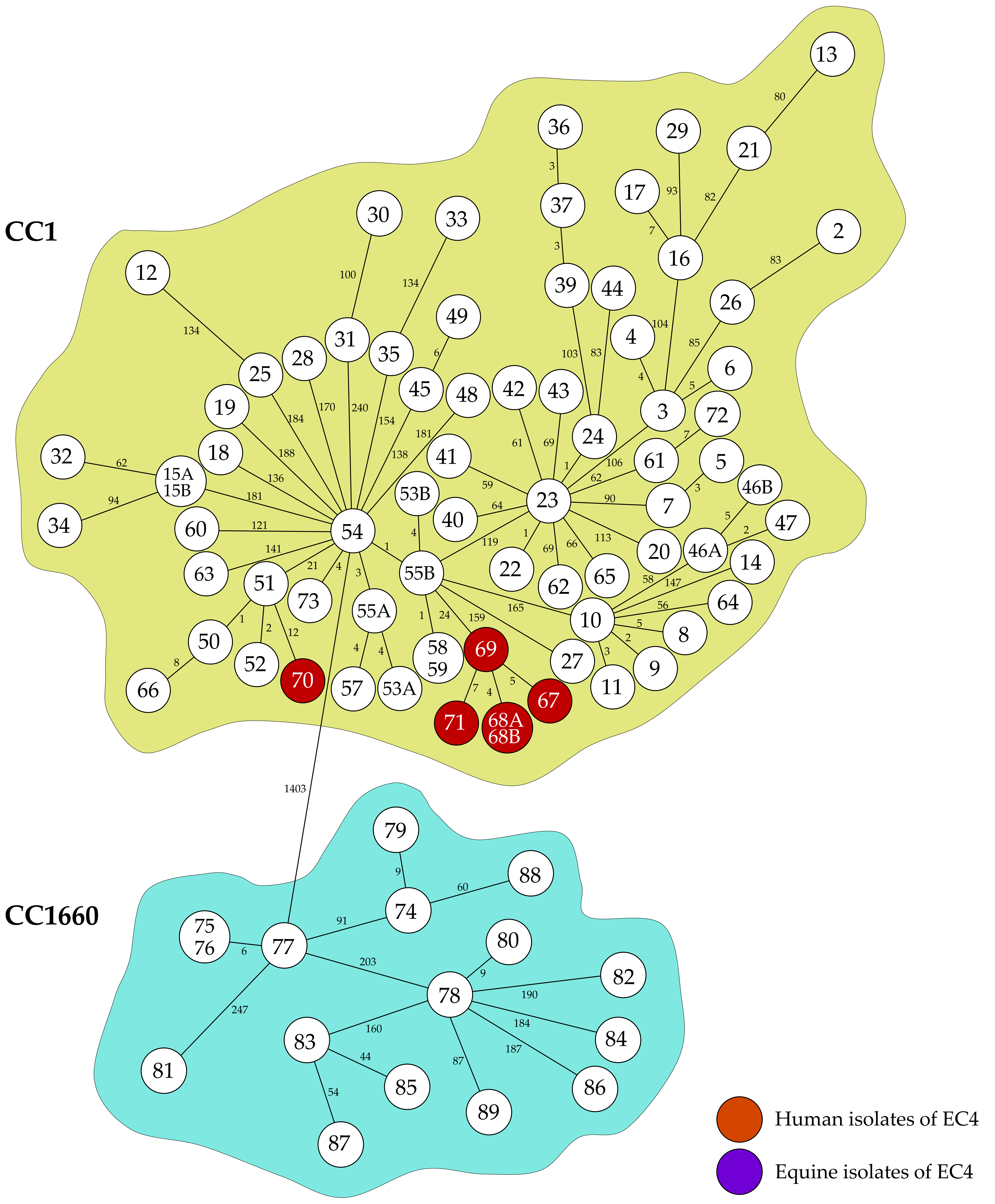

e)

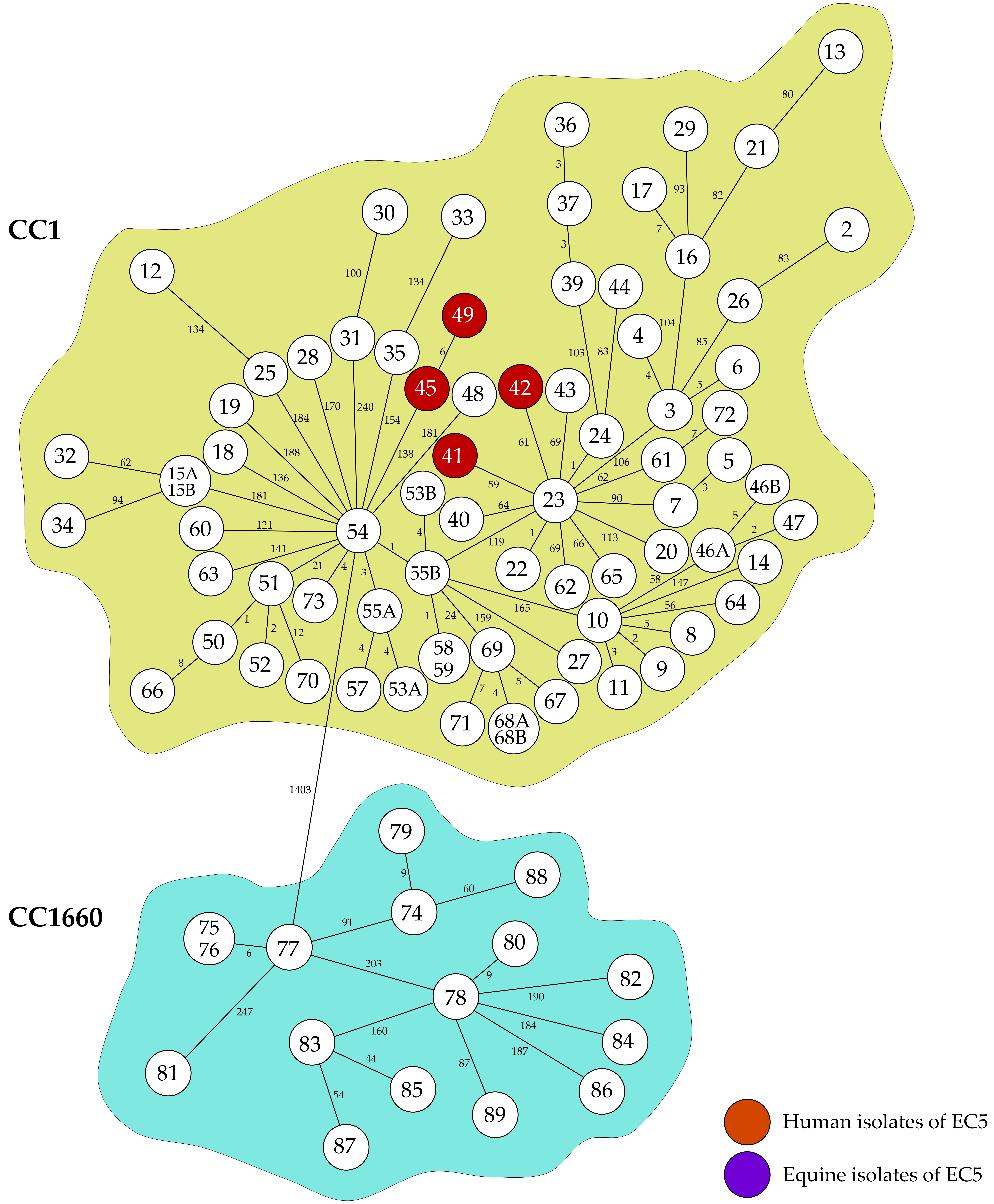

f)

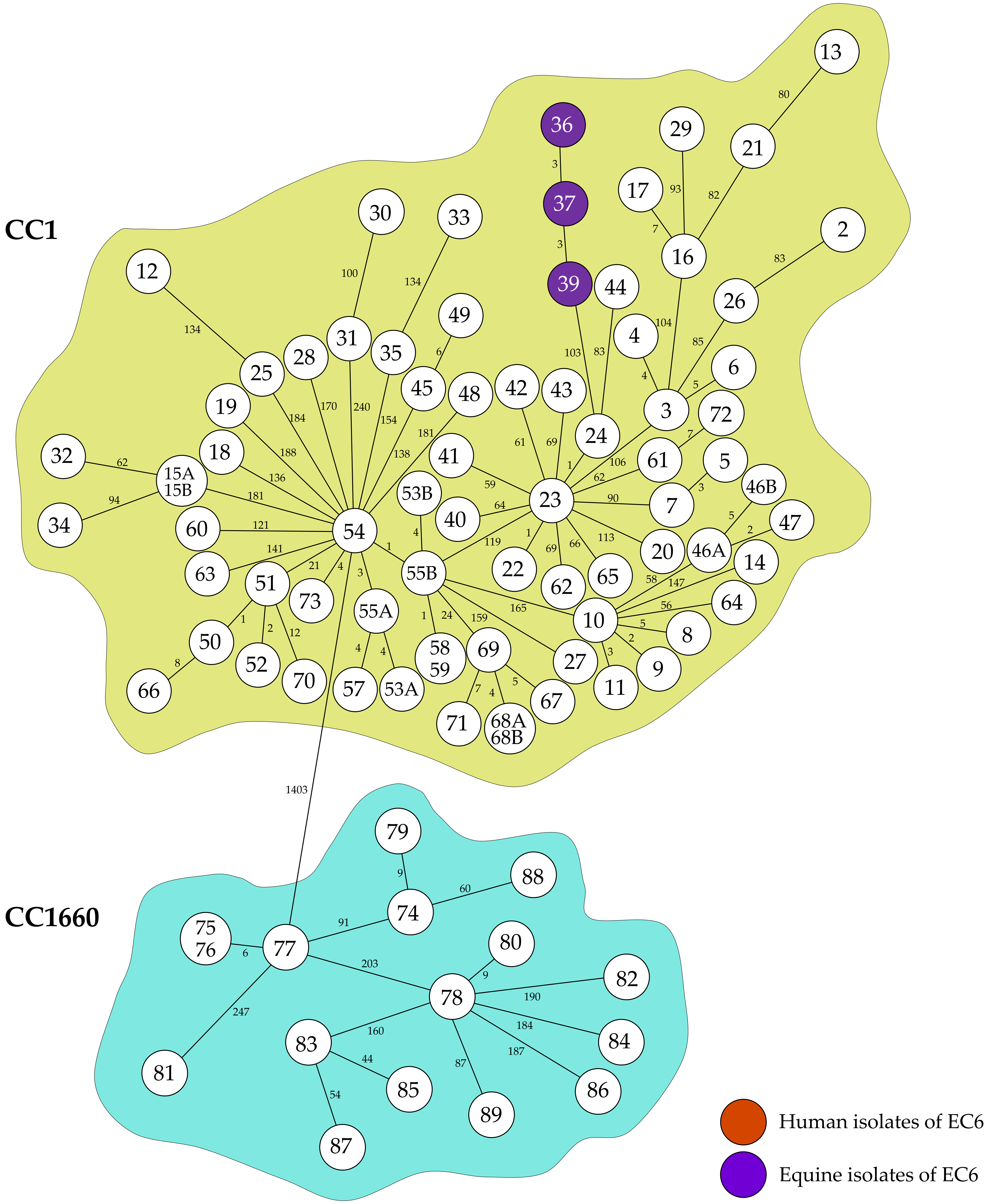

g)

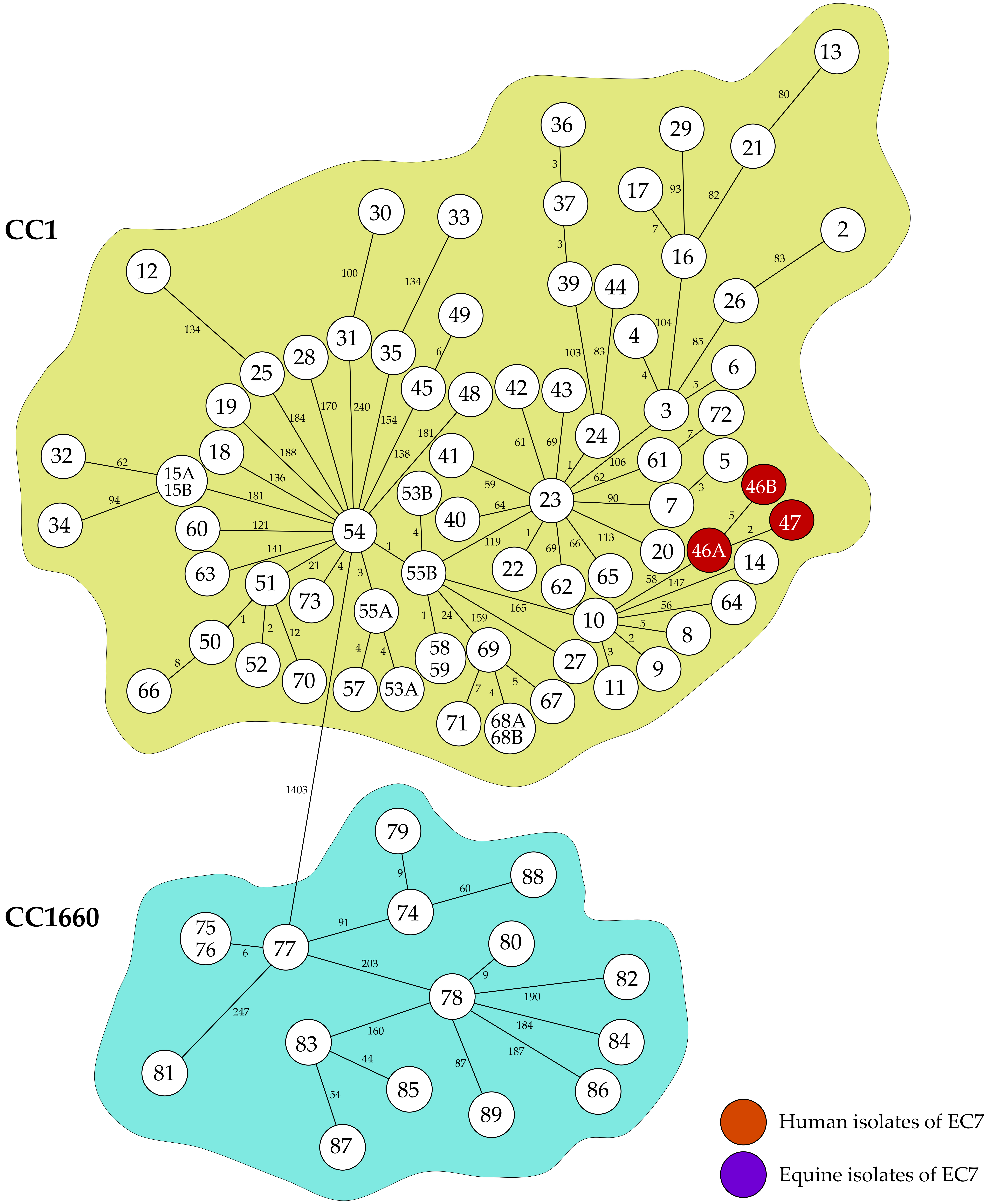

h)

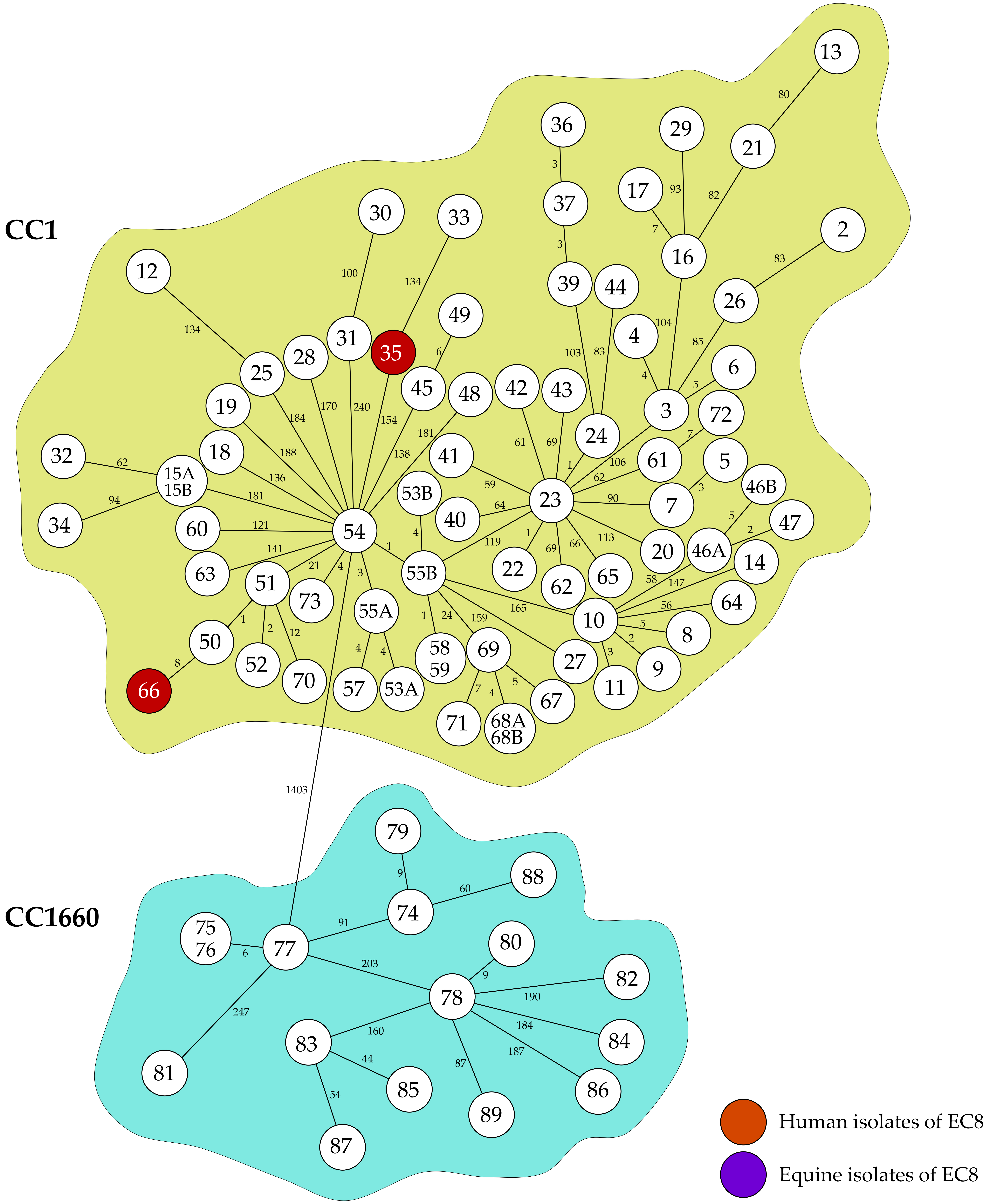

i)

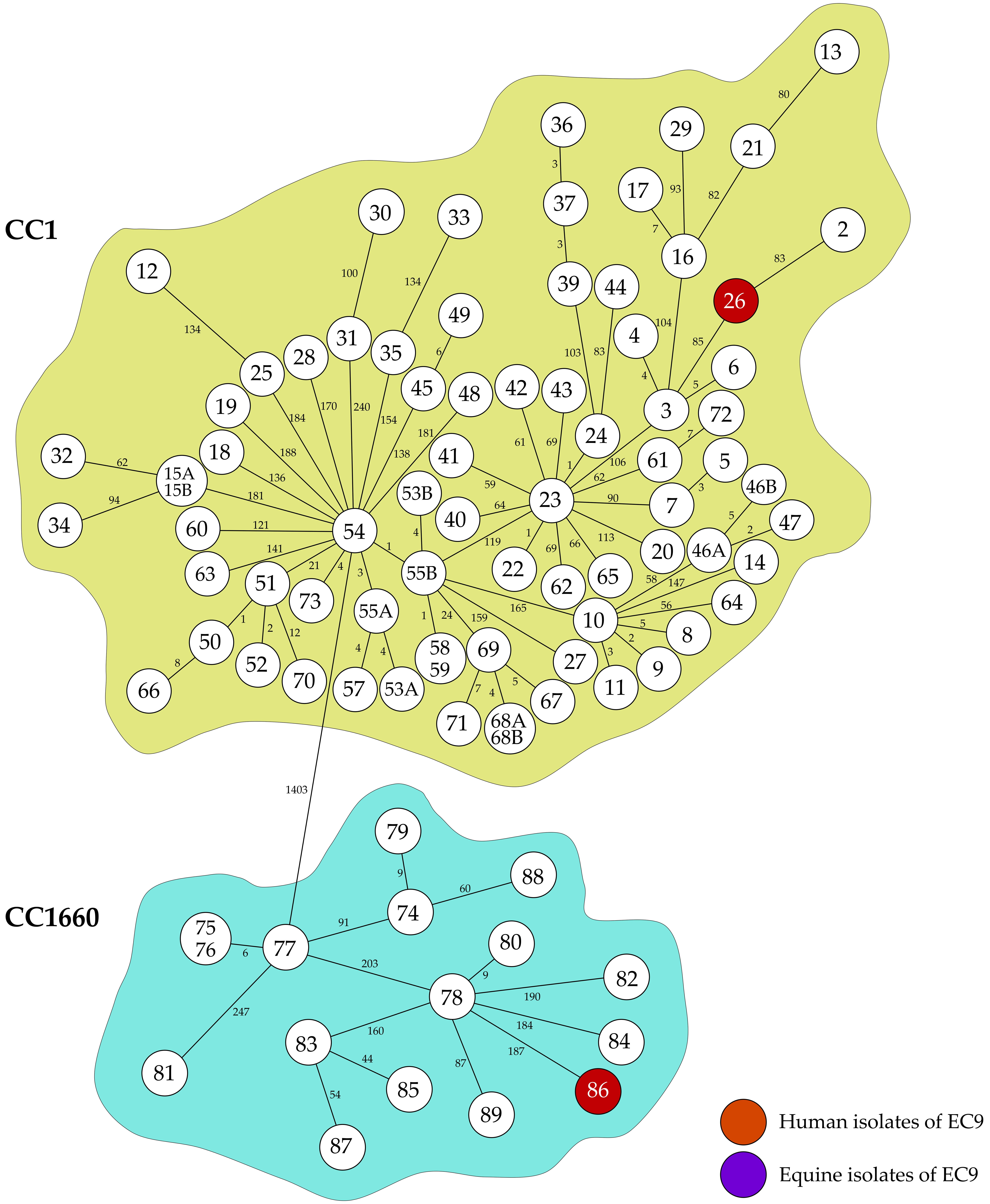

Figure S3. Distribution of the isolates from the different equine clinics (EC)

(a) EC1, (b) EC2, (c) EC3, (d) EC4, (e) EC5, (f) EC6, (g) EC7, (h) EC, (i) EC9

Minimum spanning tree based on the cgMLST allelic profile of the 91 *S. aureus* isolates (1492 columns; 1861 columns for distance calculation, 369 columns with missing values in at least one sample removed). The distance between isolates is indicated by the number next to the line, length of line not proportionate. The correlation of numbers and isolate IDs is given in table S1.

a) *spa* types from CC1

t114:	07	-	-	-	-	-	-	16	34	33	13
t127:	07	23	21	-	-	-	-	16	34	33	13
t273:	07	23	21	17	13	34	-	16	34	33	13
t559:	07	23	21	-	-	-	-	-	-	-	13
t922:	07	23	21	-	-	-	-	16	-	33	13
t1383:	07	23	21	-	-	-	-	-	-	33	13
t1491:	07	23	21	17	13	34	34	16	34	33	13
t1508:	15	-	-	-	-	-	-	16	34	33	13
t18599:	15	-	-	-	-	-	-	16	02	-	13
t14504:	26	23	21	-	-	-	-	16	34	31	13

r07: GAGGAAGACAACAACAAACCTGGT
r15: GAGGAAGACAACAACAAGCCTGG**C**
r26: GAGGAAGACAACAA**A**AAACCTGGT

r34: AAAGAAGACAACAAAAAACCTGGT
r02: AAAGAAGACAACAAAAAACCTGG**C**

r33: AAAGAAGATGGCAACAAGCCTGGC
r31: AAAGAAGATGGCAACAA**A**CCTGGC

b) *spa* types from CC1660

t549:	04	20	69	31	70	13	17	16	16	16		
t3043:	04	20	69	31	70	13	17	16	16	16	16	
t11926:	04	20	69	25	-	-	-	16				
t12047:	04	20	69	31	70	13	17	16	16	16	16	16
t15977:	04	20	69	31	70	13	17	16	17	16		

r25: AAAGAAGATGGCAACAAACCTGGT
r31: AAAGAAGATGGCAACAAACCTGG**C**

r16: AAAGAAGACGGCAACAAACCTGGT
r17: AAAGAAGACGGCAACAA**G**CCTGGT

Figure S4. Genetic relationship of the *spa* types detected among the CC1 (a) and CC1660 (b) isolates.
For each CC the different repeats of the *spa* type are indicated in different colors. Differences between different repeats present in the same position are indicated in red and bold in the compared repeats.

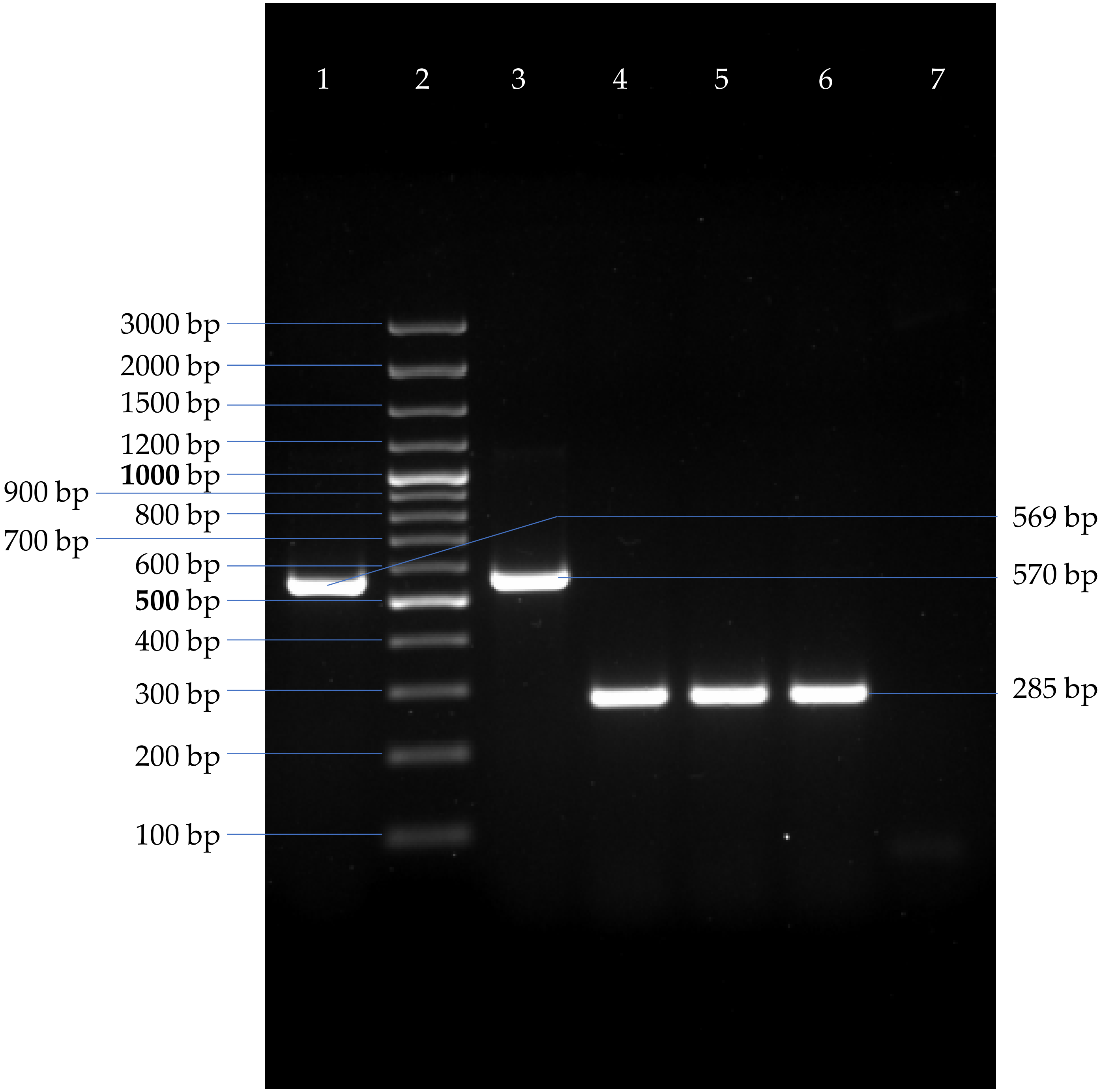

Figure S5. ***arcC*-PCR**

Lane 1: 13-03859-1660.7
Lane 2: GeneRuler® 100pb Plus Ladder
Lane 3: positive control (18-02441-40)
Lane 4: 18-02051-36
Lane 5: 18-02052-37
Lane 6: 18-02054-39
Lane 7: negative control

a)

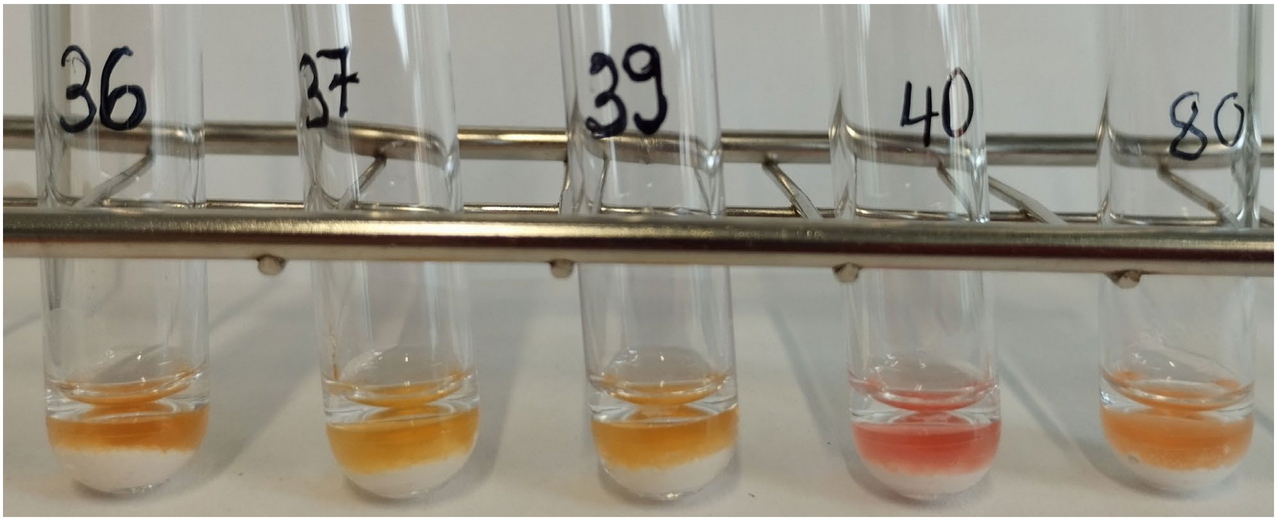

b)

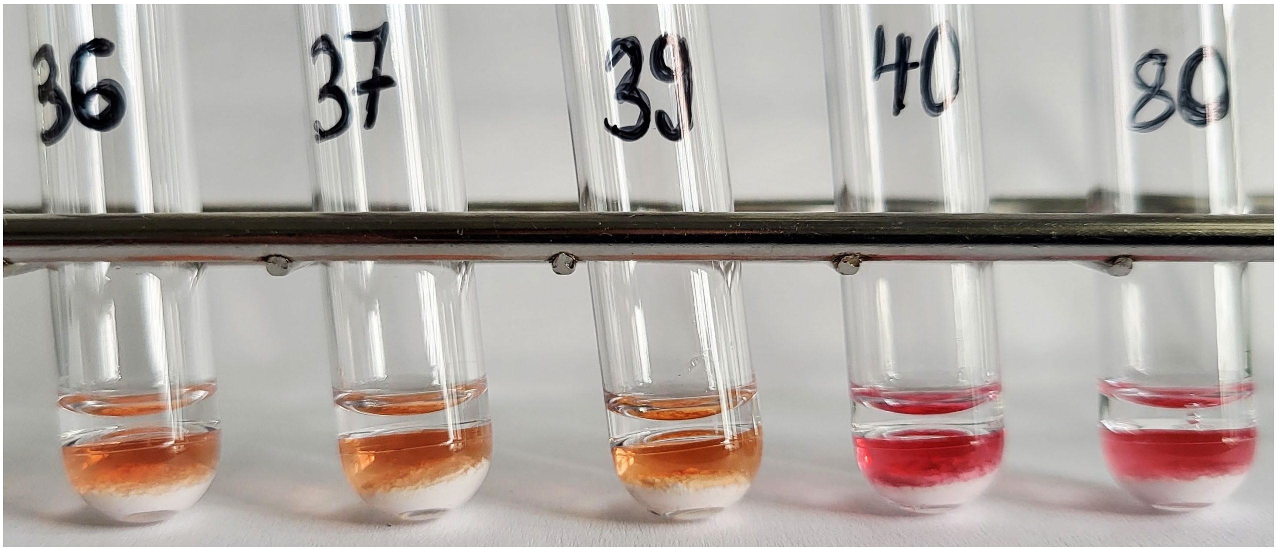

Figure S6. Testing of the anaerobic arginine dehydrolase activity
Evaluation after 4 h (a) and 24 h (b) of incubation at 37°C.
The tested isolates are indicated as follows:
95 bp deletion: 36 (18-02051-36), 37 (18-02052-37), and 39 (18-02054-39) ,
wild type *arc*C, positive control: 40 (18-02441-40)
1 bp deletion: 80 (13-03859-1660.7)
pink = positive, yellow negative
